# Amine‐Functionalized Triazolate‐Based Metal–Organic Frameworks for Enhanced Diluted CO_2_ Capture Performance

**DOI:** 10.1002/anie.202424747

**Published:** 2025-01-31

**Authors:** Klara Klemenčič, Andraž Krajnc, Andreas Puškarić, Matej Huš, Dana Marinič, Blaž Likozar, Nataša Zabukovec Logar, Matjaž Mazaj

**Affiliations:** ^1^ National Institute of Chemistry Hajdrihova 19 1000 Ljubljana Slovenia; ^2^ University of Nova Gorica Vipavska cesta 13 5000 Nova Gorica Slovenia; ^3^ Rudjer Bošković Institute Bijenička cesta 54 10000 Zagreb Croatia; ^4^ Association for Technical Culture of Slovenia (ZOTKS) Zaloška 65 1000 Ljubljana Slovenia; ^5^ Institute for the Protection of Cultural Heritage (ZVKDS) Poljanska 40 1000 Ljubljana Slovenia; ^6^ Faculty of Chemistry and Chemical Engineering University of Maribor Smetanova 17 2000 Maribor Slovenia

**Keywords:** diluted CO_2_ capture, amino-functionalized MOFs, wet CO_2_ adsorption, indoor air purification, solid-state NMR analysis

## Abstract

Efficient CO_2_ capture at concentrations between 400–2000 ppm is essential for maintaining air quality in a habitable environment and advancing carbon capture technologies. This study introduces NICS‐24 (National Institute of Chemistry Structures No. 24), a Zn‐oxalate 3,5‐diamino‐1,2,4‐triazolate framework with two distinct square‐shaped channels, designed to enhance CO_2_ capture at indoor‐relevant concentrations. NICS‐24 exhibits a CO_2_ uptake of 0.7 mmol/g at 2 mbar and 25 °C, significantly outperforming the compositionally related Zn‐oxalate 1,2,4‐triazolate – CALF‐20 (0.17 mmol/g). Improved performance is attributed to amino‐functions that enhance CO_2_ binding and enable superior selectivity over N_2_ and O_2_, achieving 8‐fold and 30‐fold improvements, respectively, in simulated CO_2_/N_2_ and CO_2_/O_2_ atmospheric ratios. In humid environments, NICS‐24 retained structural integrity but exhibited an 85 % reduction in CO_2_ capacity due to competitive water adsorption. Breakthrough sorption experiments, atomistic NMR analysis, and DFT calculations revealed that water preferentially adsorbs over CO_2_ due to strong hydrogen‐bonding interactions within the framework. Gained understanding of the interaction between CO_2_ and H_2_O within the MOF framework could guide the modification via rational design with improved performance under real‐world conditions.

## Graphical abstract

NICS‐24, a novel Zn(II)‐based diaminotriazolate oxalate MOF, exhibits significant improvements in CO_2_ adsorption at low concentrations compared to compositionally related CALF‐20. In humid conditions, competitive adsorption of water and CO_2_ occurs, facilitated by hydrogen bonding interactions between the framework and guest molecules, as well as between the guest molecules themselves.

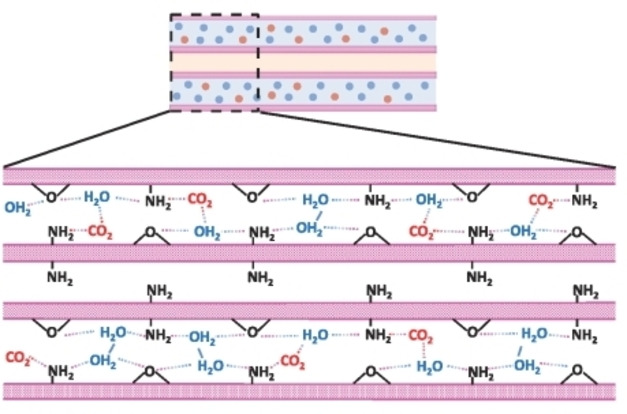

## Introduction

Indoor air quality stands as a cornerstone of public health, with profound implications for human well‐being. A significant aspect of maintaining indoor air quality involves the capture and management of carbon dioxide (CO_2_), particularly under conditions where it accumulates in inhabited environments due to human metabolism. This is especially critical in spaces lacking efficient ventilation systems. Elevated CO_2_ levels, with a typical threshold concentration of 2000 ppm, can have negative impacts on human health, causing headaches and fatigue, which can escalate to nausea, dizziness, vomiting, and even death as CO_2_ concentrations increase.[^1^, ^2^, ^3^] Repercussions of such elevated CO_2_ levels necessitate effective strategies for its capture and management, making this a pressing concern.

Simultaneously, the technological demand for pure CO_2_ is increasing rapidly, yet the availability of energy‐efficient processes to provide adequately pure CO_2_ gas for various applications remains limited.[^4^, ^5^, ^6^] In this context, the utilization of advanced solid adsorbents, particularly through physisorption, emerges as a promising solution to tackle these intertwined challenges.[^7^, ^8^]

Metal–organic frameworks (MOFs) offer great potential to mitigate these challenges due to their high flexibility in tuning the structure–property relationship by rational design.[^9^, ^10^] However, the stringent requirements for indoor CO_2_ capture, including ambient pressure and temperature conditions, selective adsorption at diluted CO_2_ concentrations, and performance resilience in the presence of humidity, drastically narrow down the selection of suitable MOF candidates.[^11^, ^12^, ^13^, ^14^] The overall CO_2_ sorption capacity is primarily determined by the strength of CO_2_ sorption at low pressures and the available free pore volume at higher pressures.^[15]^ It is already known that the most suitable pore size for CO_2_ storage with optimal CO_2_‐to‐MOF interaction is between 4–7 Å, and that the presence of polar functional groups and specific metal centers significantly enhance the affinity towards CO_2_ sorption.[^16^, ^17^] Considering practical indoor air CO_2_ application it is imperative that efficient selective capture can be performed at room temperature both at higher and low pressures. MOF‐74(Mg) is one of the most frequently investigated material for CO_2_ capture reaching the adsorption capacity of 6.3 mmol/g of adsorbent (27.5 wt %) up to 1 bar at 25 °C with some other MOF systems such as UTSA‐16 or UiO‐66 exhibiting comparable CO_2_ capture performances.[^18^, ^19^, ^20^] Precise tailoring of the pore size using of pyrazine‐based ligands, along with the introduction of electrostatic SiF_6_^2−^ and (NbOF_5_)^2−^ anions, yielded the SIFSIX and NbOFFIVE families, respectively, exhibiting single‐component adsorption capacities up to 2.2 mmol of CO_2_/g at 400 ppm partial pressure (NbOFFIVE‐1).[^21^, ^22^] A new generation of Anion‐Pillared MOFs (APMOFs) is also being reported for benchmark CO_2_ capture with customized cages.^[23]^ A breakthrough in the design of MOFs for direct air capture technologies was achieved with the *N*,*N’*‐dimethylethylene diamine (MMEN) modification of Mg_2_(dobpdc) (dobpdc=4,4’dioxidobiphenyl‐3,3’dicarboxylate), due to cost‐efficient production and achieving CO_2_ adsorption capacity of 3.0 mmol/g at direct air capture conditions, which is beyond the capture capacity criteria for such technologies.^[24]^

However, in many cases, the co‐adsorption of water in a humid environment can significantly reduce the adsorption capacity for CO_2_, highlighting the importance of understanding and optimizing the adsorption mechanisms.^[25]^ Notably, the presence of moisture complicates the adsorption, particularly in amino‐functionalized MOFs. While competitive adsorption of water can significantly reduce the CO_2_ capture capacity at low partial pressures, certain studies have shown that moisture can enhance CO_2_ uptake due to its influence on the adsorption mechanism. For instance, under dry conditions, the formation of carbamate involves two amine groups capturing one CO_2_ molecule.[^26^, ^27^, ^28^] However, under humid conditions, water can facilitate the formation of bicarbonate ions, requiring only one mole of amine per mole of CO_2_, thus increasing the adsorption capacity. Moreover, hydronium carbamate formation and weak hydrogen bonds between hydronium carbamate and bicarbonate have also been identified as contributing factors to enhanced CO_2_ capture in humid environments.^[29]^

Among the notable advancements, the Zn‐triazolate oxalate structure (CALF‐20) signifies a breakthrough in post‐combustion CO_2_ capture, owing to its facile synthesis and high capture performance even in humid conditions.^[30]^ Nevertheless, its sorption capacities and selectivity for CO_2_ diminish at concentrations below approximately 5 % due to weak binding energies. Removing CO_2_ from indoor air, however, requires efficient capture at even lower partial pressures (around 2 mbar). It has been shown that the presence of amine groups in a 3‐amino‐1,2,4‐triazolate structure analogue (CALF‐15) enhances affinity for CO_2_ binding and promotes selective adsorption at lower partial pressures of CO_2_.^[31]^

Recognizing the potential of amino‐functionalized MOFs to enhance CO_2_‐to‐framework interactions even further, our focus shifts to the design of new guanazole (3,5‐diamino‐1,2,4‐triazole)‐based oxalate MOF, designated as NICS‐24. By increasing the concentration of amine groups within the MOF framework, we aim to enhance the propensity for CO_2_ adsorption at highly diluted concentrations, addressing a critical gap in the capabilities of existing materials like CALF‐20, which is a benchmark for post‐combustion CO_2_ capture. Furthermore, we rigorously evaluate the role of water on CO_2_ adsorption performance through breakthrough experiments and solid‐state NMR experiments supported by computational approaches to gain a deeper understanding of the underlying adsorption mechanisms.

## Results and Discussion

**Figure 1:**
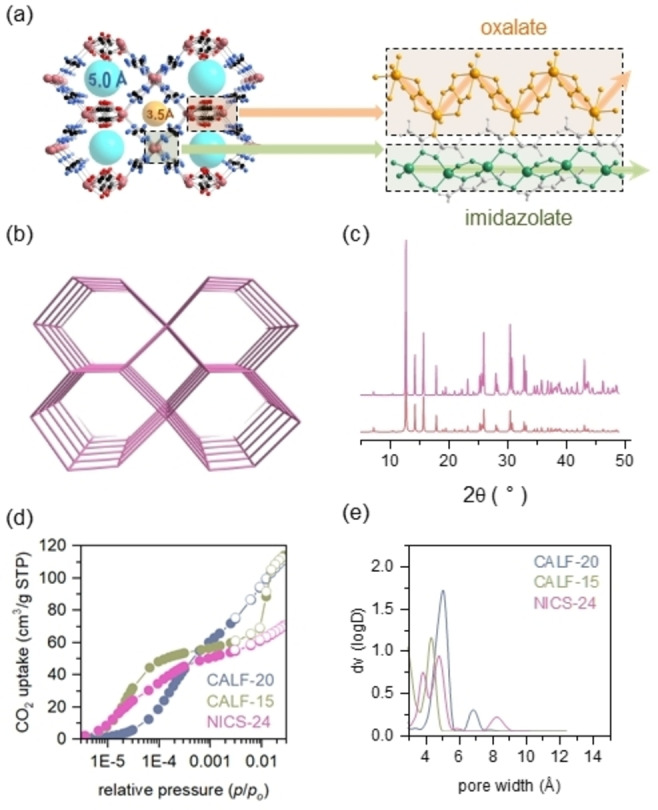
(a) Left ‐ NICS‐24 framework oriented along (001) direction. Spheres located within the channels symbolize free space with the indicated pore diameter estimated geometrically between opposite atoms defining the channel boundaries. Zn – purple circles, N – blue circles, C – black circles. Right – location of two type of Zn‐centered chains – zigzag oxalate‐based (ochre) and straight imidazolate‐based (green) chain. Atoms which are not involved in Zn(II) connectivity are colored in light grey. (b) Simplified representation of NICS‐24 framework indicating *sqc* topology. (c) XRD pattern of bulk NICS‐24 sample (top) compared to the calculated pattern (below). (d) CO_2_ isotherms of CALF‐20, CALF‐15 and NICS‐24 measured at 273 K (full circles – adsorption points, empty circles – desorption points) with (e) corresponding NLDFT pore size distribution profiles.

NICS‐24 was synthesized using various conventional methods, each carefully designed to demonstrate the material‘s versatility and ease of production (experimental details in SI, Figures S1–S3). The straightforward nature of the synthesis enables scalability and the potential for fine‐tuning the material's properties, being crucial for advancing from laboratory research to real‐world deployment. NICS‐24 structure (Zn‐oxalate 3,5‐diamino‐1,2,4‐triazolate) determined from powder XRD data (Tables S1–S4) exhibits two crystallographically distinct Zn(II) centers (Figure S4). Zn1 is in a distorted octahedral coordination environment, connected with four oxygen atoms from two oxalate anions and two nitrogen atoms from bridging diaminotriazolate ligand. Zn2 is in tetrahedral environment coordinated solely to triazole nitrogen atoms. Each type of zinc centers forms specific type of infinite chains assigned as oxalate zigzag chains for Zn1 and triazolate straight chains for Zn2 (Figure 1a and S5). Both chains are bridged with diaminotriazolate ligands resulting in generation of two types of square‐shaped channels along [001] direction with the dimensions of 3.5 Å (type A) and 5.0 Å (type B) and formation of 3D framework with *sqc* topology (Figure 1b). The bulk product corresponds to the described structure (Figure 1c, S6).

In order to study the effect of amine functions on diluted CO_2_ capture applications, structural features, textural properties and CO_2_ sorption performances of NICS‐24 were readily compared with compositionally related Zn(II)‐1,2,4‐triazolate oxalate (CALF‐20) and Zn(II)‐3‐amine‐1,2,4‐triazolate oxalate (CALF‐15) (synthesis details in Supporting Information and Figure S7). CALF‐20 structure, previously elucidated by Shimizu et al.,^[30]^ comprises of Zn‐oxalate chains linked by 1,2,4‐triazolate ligands oriented in two directions. These ligands form intersecting square‐shaped channels with the estimated dimensions of 4.5×4.7 Å and 4.6×5.4 Å along the [100] and [011] directions respectively. Its isostructural analogue, CALF‐15, features 3‐amino‐functionalized triazolate ligands with slightly narrower pore dimensions compared to CALF‐20 (3.8×4.4 Å and 3.8×3.9 Å along [100] and [011] directions, respectively) due to the amine groups oriented inside [011] channels.[^31^, ^32^] Structure schemes of investigated materials are represented in Figure S8.

All three structures exhibit consistent thermal stability, with thermogravimetric analysis (Figure S9) indicating ligand decomposition occurring within the temperature range of 300–400 °C. Particularly, NICS‐24 maintains its framework structure with only slight change of unit cell parameters upon drying up to 250 °C which shows high framework rigidity before decomposition (Figure S10, Table S5). To assess porosity properties, all three products (CALF‐20, CALF‐15 and NICS‐24) were activated using the same two‐step degassing protocol i.e. 2 h at 60 °C and 10 h at 100 °C.^[30]^ Notably, N_2_ adsorption at 77 K, yielded expected BET surface area and micropore volume values solely for the CALF‐20 material. Conversely, CALF‐15 and NICS‐24 displayed negligible N_2_ uptakes despite possessing comparable pore dimensions and geometries to CALF‐20 (Figure S11). Lower N_2_ sorption capacities as expected are likely attributed to the extremely slow adsorption kinetics at cryo‐conditions, hindering N_2_ diffusion through ultra‐micropore channels.[^33^, ^34^] Porosity properties were therefore further evaluated using CO_2_ sorption isotherms measured at 273 K for all samples to ensure consistency (Figures 1d, S12 and S13). CALF‐20 exhibits Langmuir‐type isotherm consistent with previously published data.^[30]^ It gradually approaches saturation with a final CO_2_ uptake of 112 cm^3^/g STP up to 1 bar resulting in BET surface area of 402 m^2^/g and a relatively broad pore size distribution with the NLDFT profile peak positioned at 0.53 nm. CALF‐15 on the other hand displays Type I isotherm, reaching saturation above p/p_0_=0.005, exhibiting pore size distribution with the peak at 0.39 nm. BET surface area is notably lower compared to CALF‐20 (314 m^2^/g) due to narrower pores. Material demonstrates gradual gate opening effect, reflecting in significant increase of CO_2_ adsorption in the p/p_0_ pressure range between 0.01–0.015.^[35]^ NICS‐24 exhibits similar Type I isotherm as CALF‐15 with slightly lower equilibrium uptake at p/p_0_=0.005 (63 cm^3^/g STP and 57 cm^3^/g STP for CALF‐15 and NICS‐24 respectively), resulting in a BET surface area of 274 m^2^/g. Bimodal pore size distribution with NLDFT profile peaks at 0.36 nm and 0.49 nm coincides with the structure possessing two types of channels (Figures 1e and S14).

Capture performance at diluted CO_2_ concentrations were evaluated for NICS‐24 and compared with CALF‐20 and CALF‐15 materials using their adsorption capacity, selectivity, binding energy, regeneration capability, durability and capture performance in humidity.

**Figure 2:**
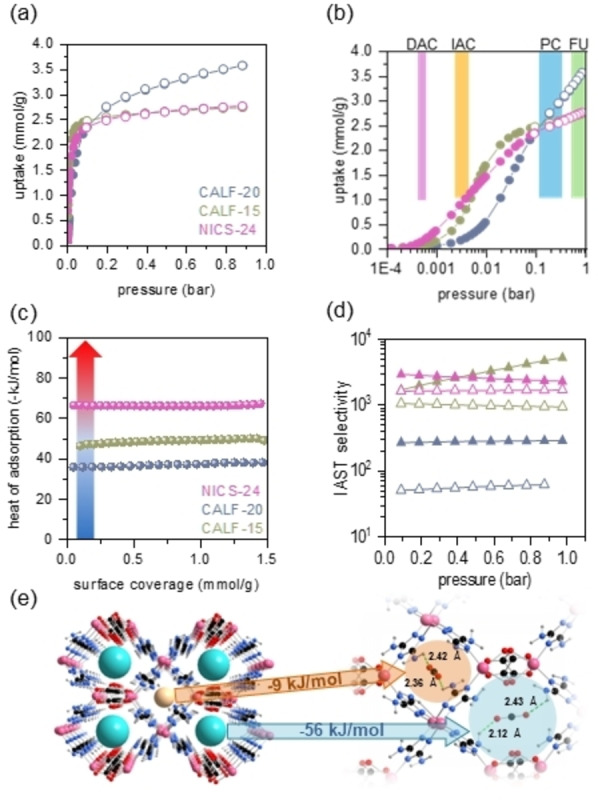
(a) CO_2_ isotherms measured at 25 °C for NICS‐24, CALF‐15 and CALF‐20 (full circles – adsorption points, empty circles – desorption points). (b) CO_2_ isotherms measured at 25 °C plotted in logarithmic scale. Coloured areas indicate pressure regions relevant for specific CO_2_ capture application; BFU – biofuel upgrade, PCU – post‐combustion capture and utilization, IAC – indoor‐air capture, DAC – direct air capture. (c) Isosteric heat of adsorption values for CO_2_ for NICS‐24, CALF‐15 and CALF‐20. Gradient‐coloured arrow indicates binding type from blue – physisorption, to red – chemisorption; (d) IAST selectivity for NICS‐24, CALF‐15 and CALF‐20. CO_2_/N_2_ at 0.2 % of CO_2_ – full symbols, CO_2_/O_2_ at 0.8 % of CO_2_ – empty symbols. (e) Preferential binding sites for CO_2_ within both types of NICS‐24 channels with the indicated interatomic distances between H‐atom originated from diaminotriazole amine group and O‐atom from CO_2_ molecule (green dashed line) and calculated heat of adsorption values for specific site given within arrows.

Isotherms of pure gas at specific temperature and pressure regions are commonly employed as a primary screening tool to assess absolute CO_2_ capture capacity. However, the values obtained from such assessments do not fully reflect the true capture performance, since the working conditions such as partial pressure region, selectivity and kinetics are being neglected in that case.^[36]^ Single‐component adsorption capacity is generally influenced by accessible pore volume and BET surface area, though chemical functionality and pore environment also play crucial roles, as seen in differences between materials like activated carbons, zeolites, and MOFs. Indeed, CALF‐20 with the highest surface area value among investigated materials, demonstrates the highest sorption capacity at 1 bar and 25 °C, reaching 4.1 mmol/g (Figure 2a). CALF‐15 and NICS‐24 adsorb similar amounts of CO_2_ (3.0 mmol/g) under these conditions. However, for specific CO_2_ capture application (Figure 2b), the capacity at desired partial pressures holds more relevance than the total capacity.[^7^, ^37^, ^38^, ^39^] At 0.15 bar (post‐combustion CO_2_ capture—PC), CALF‐20 continues to demonstrate superiority with a CO_2_ uptake of 2.9 mmol/g compared to CALF‐15 and NICS‐24, which adsorb 2.6 and 2.4 mmol of CO_2_/g of adsorbent, respectively. However, the sorption capacity of CALF‐20 and CALF‐15 decrease significantly below the ‘practical threshold’ at 1 mmol/g when the partial pressure of CO_2_ is lowered to 2 mbar (indoor air capture—IAC). Conversely, NICS‐24 still holds CO_2_ uptake of 0.7 mmol/g under such conditions with the sorption capacity dropping below 1 mmol/g when the partial pressure decreases to 1 mbar (Figure 2b). The observed capacity rates NICS‐24 within the broader context of amine‐appended MOF materials engineered for low concentration CO_2_ capture (Table S6). Additionally, material shows high durability over 20 adsorption/desorption cycles using temperature‐swing adsorption regeneration (Figure S15). Single‐component adsorption isotherms so suggest the potential suitability of NICS‐24 for diluted CO_2_ capture applications. The reproducibility of CO_2_ sorption performance for NICS‐24 was assessed by measuring CO_2_ isotherms across several product batches (Figure S16). The observed variability in uptake values, approximately 20 % at concentrations up to 2000 ppm, demonstrates the robustness and consistency of the material's performance.

CO_2_‐to‐framework interaction energy (*Qst*) is a critical factor influencing the adsorption capacity, selectivity and the energy efficiency of regeneration processes. The optimal heat of adsorption is estimated to range between 30 and 60 kJ/mol.^[40]^ This range balances strong binding for highly selectivity with the ability to release CO_2_ under mild conditions. Notably, optimal range may vary with CO_2_ concentration in the stream, with more diluted CO_2_ requiring higher enthalpy values for effective capture and release cycles. In this study, *Qst* values were calculated using Clausius‐Clapeyron equation based on isotherms measured between 25 and 35 °C (Figures 2c, S17–S20 and Table S7).^[41]^ For CALF‐20, the *Qst* ranges from 29 and 37 kJ/mol at low surface coverages (up to 1 mmol/g), aligning with the theoretical value of 36.5 kJ/mol predicted by DFT.^[30]^ This heat of adsorption value reflects modest physisorption, driven predominantly by van der Waals interactions. In contrast, Zn‐aminotriazole oxalate (CALF‐15) exhibits significantly higher *Qst* values between 49 and 53 kJ/mol, attributed to enhanced electrostatic interactions and spatial confinement effects within its reduced pore size. NICS‐24, containing two amine groups on triazole linker, further increases the *Qst* to 68 kJ/mol. This value approaches the upper limit for energy‐efficient regeneration particularly for temperature swing processes (TSA)^[24]^ and highlights the role of amine‐functionalized frameworks in enhancing CO_2_‐framework interactions. While the framework achieves stronger binding, the increased heat of adsorption may reflect in higher energy costs for desorption even though the interactions are still considered to be of physisorptive nature. The progression of Q*st* values across CALF‐20, CALF‐15, and NICS‐24 demonstrates the interplay of pore size, amine functionality, and framework design in tailoring CO_2_ capture performance. By synchronizing weak physisorption interactions with structural confinement effects, these materials strike varying balances between adsorption strength and regeneration efficiency.

High sorption selectivity for CO_2_ enables delivery of the captured gas in high purity, which is pivotal for applications where CO_2_ utilization is targeted. Moreover, in indoor environments, selective CO_2_ capture prevents removal of components essential for maintaining life support systems. Sorption selectivity for CO_2_ against N_2_ and O_2_, the most abundant gas components in the atmosphere, was evaluated using IAST^[42]^ considering CO_2_ concentrations between 400 and 4000 ppm (Figures 2d and S21–S29). Since the partial pressure of O_2_ in the atmosphere is typically four times lower than of N_2_, CO_2_/O_2_ ratios were adapted accordingly in the selectivity calculations. CALF‐20 exhibits high CO_2_/N_2_ selectivity at low CO_2_ concentrations, primarily due to dominant dipol‐quadropole interactions between CO_2_ and triazolate ligands.^[43]^ However, the presence of amine groups in CALF‐15 and NICS‐24 further enhances the selectivity. This is attributed to the narrower pores in CALF‐15 and NICS‐24 compared to CALF‐20, which favor CO_2_ adsorption over N_2_, and increased interactions between CO_2_ molecule and amine groups. A similar trend of selectivity enhancement is observed for CO_2_/O_2_ (Figure 2d). Notably, lower selectivity values for oxygen compared to nitrogen in CALF‐20 and CALF‐15 can be attributed to the smaller kinetic diameter of O_2_ (3.46 Å vs 3.64 Å respectively), allowing better diffusion of O_2_ over N_2_, and the quadrupole moment of O_2_ which may lead to slightly stronger interactions with the framework compared to nitrogen molecule. On the other hand, CO_2_/N_2_ and CO_2_/O_2_ selectivity for NICS‐24 are more comparable, implying that steric hindrance impedes O_2_ from accessing adsorption sites almost as effectively as N_2_ in ultramicroporous channels of NICS‐24.

Theoretical calculations reveal two possible adsorption sites for CO_2_, in NICS‐24, corresponding to two pore types, as shown in Figure 2e. The calculated adsorption energy in the B and A channels are 56 and 9 kJ/mol, respectively. These values align well with the experimentally determined heat of adsorption of 63 kJ/mol, indicating a dominant physisorption with localized interactions. In type B channel, CO_2_ assumes a transversal position, approximately equidistant from the pore walls. It interacts weakly with the adjacent amino groups of the 3,5‐diamino‐1,2,4‐triazolate scaffolding. The OC−O…HN(H)− distance is 2.35 Å on both sides of the pore, typical of physisorptive interactions. Bader charge analysis confirms minimal charge transfer. In isolated CO_2_, the charges on C and O are +1.96 e_0_ and −0.98 e_0_, respectively, which are increased to +2.00 e_0_ and −1.00 e_0_ when bound in NICS‐24. In channel A, CO_2_ would theoretically orient along the dimension of the pore with the OC−O…HN(H)− distance of 2.3–2.4 Å. However, due to the very low adsorption energy (−9 kJ/mol), such positioning does not occur. The upper limit of NICS‐24 capacity was determined by filling both pores with CO_2_. Dependent on the CO_2_ activity, the material can uptake further CO_2_ if the differential adsorption energy is favorable, i.e. negative. This changes upon the addition of the fifth CO_2_ molecule per unit cell—the first one to occupy the small pore, which puts the maximum uptake at 2.4 mmol/g which aligns perfectly with the isothermal measurements.

Most CO_2_ capture processes involve exposure to water vapor, a highly competitive component that can significantly impact CO_2_ capture efficiency. Preferential binding of water molecules to the sorption sites or ligand displacement can drastically reduce the overall sorption performance for CO_2_ and poses a risk of gradual degradation to the MOF structures, potentially compromising their long‐term stability and functionality.[^11^, ^44^]

**Figure 3:**
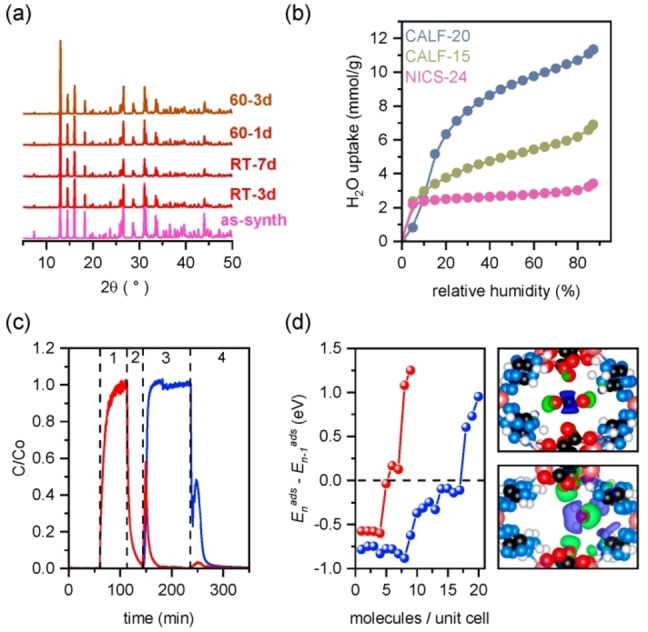
(a) XRD patterns of pristine NICS‐24 (as‐synth) and after soaking in water at room temperature for 3 days (RT‐3d), 7 days (RT‐7d) and at 60 °C after 1 day (60‐1d) and 3 days (60‐3d). (b) Water adsorption isotherms of CALF‐20, CALF‐15 and NICS‐24 measured at 25 °C. (c) Breakthrough curves showing the effect of humidity on CO_2_ adsorption/desorption dynamics (m/z CO_2_ signal – red, m/z H_2_O signal – blue. 1) saturating the sorbent with 2000 ppm CO_2_, 2) purging the system with He to remove physically bound CO_2_, 3) water injection to the purge gas causing CO_2_ desorption and 4) temperature swing desorption to remove remaining CO_2_. (d) Differential adsorption energy of CO_2_ (blue) and H_2_O (red) as the number of adsorbate molecules in unit cell is increased with corresponding differential electron density upon the adsorption into NICS‐24 for CO_2_ (upper right) and H_2_O (lower right) (drawn with an isosurface).

NICS‐24 exhibits high structure stability in the presence of water. Structure integrity is preserved even after soaking the material in water at 60 °C for 3 days (Figure 3a, Table S8). Water isotherms measured at 25 °C show substantial decrease of total water uptake for amino‐functionalized structures, i.e. 11.4 mmol/g, 6.9 mmol/g and 3.4 mmol/g for CALF‐20, CALF‐15 and NICS‐24, respectively (Figure 3b). The capacity trend is most probably related to the pore volume of the frameworks. However, isothermal step below 10 % RH in the case of CALF‐15 and NICS‐24 indicate that the presence of amine functional groups notably contributes to the overall hydrophilicity of the amino‐functionalized frameworks.

The effect of humidity on CO_2_ capture performance was evaluated using breakthrough curve analysis. First, He gas mixture consisting of 2000 ppm CO_2_ and 20 % O_2_ was passed through a fixed‐bed reactor at ambient temperature (Figure S30). The capacity at 25 °C in a dry environment was 0.64 mmol/g (inconsistency of CO_2_ uptakes with isothermal data is due to the difference in sorption conditions between methods). The adsorption was followed by a He purge and increasing the temperature to 120 °C to desorb physiosorbed and chemisorbed CO_2_, respectively. Physisorption was proved to be the dominant mechanism, since it contributes 57 % of the total desorption capacity. With the presence of 50 %RH, the CO_2_ capacity decreases to 0.1 mmol/g whereas the water adsorption capacity was 2.25 mmol/g. The competitive adsorption of water was additionally demonstrated by sequential adsorption experiment (Figure 3c). Upon reaching saturation of dry CO_2_ at 2000 ppm, physically adsorbed gas (0.29 mmol/g) was subsequently removed through an inert purge step. The sample then underwent purging with helium gas passing through the water vapor atmosphere resulting in final water uptake of 2.25 mmol/g. The water molecules apparently replace adsorbed CO_2_ thereby promoting the release of physisorbed CO_2_ (0.31 mmol/g). During the following desorption step, when the temperature was increased, water and remaining strongly bonded CO_2_, that remained chemically bound (0.05 mmol/g), desorbed. High stability of capture performance, along with excellent structural rigidity in humid environment, was demonstrated through breakthrough cycling, which showed consistent working capacities for CO_2_ and H_2_O over ten runs. Stability was further corroborated by XRD analysis of the product after cycling protocol (Figures S31 and S32).

Preferential binding of water vs CO_2_ in NICS‐24 was confirmed by DFT calculations. The amine groups of 3,5‐diamino‐1,2,4‐triazolate can form hydrogen bonds with water molecules, which is reflected in stronger interaction. In the larger pore, the calculated interaction energy with H_2_O is 76.3 kJ/mol, while in the smaller pore, it is 44.2 kJ/mol. In both cases, a hydrogen bond H_2_O⋅⋅⋅HN(H) is formed with an O⋅⋅⋅H distance of 1.99 Å. The strong interaction is maintained as up to eight H_2_O molecules are added within the unit cell, corresponding to a water uptake of approximately 4.4 mmol/g, which aligns well with the experimentally determined water saturation capacity (Figure 3b). Beyond this point, the differential adsorption energy gradually decays, eventually becoming slightly below 0 eV before turning positive at 17 H_2_O molecules per unit cell (∼10 mmol/g). While the theoretical calculations suggest the capacity to accommodate up to 17 water molecules, the experimentally and energetically most favourable uptake corresponds to the first 8 H_2_O molecules. Larger uptake in respect to CO_2_ is caused by two factors: stronger interaction with H_2_O on account of hydrogen bonding and smaller radius of the H_2_O molecule as compared to CO_2_. The electronic effects manifest as charge transfer. Compared to isolated H_2_O, with charges −1.22 and +0.61 e_0_ on O and H, respectively, the charge separation is increased to −1.30 e_0_ and +0.65 e_0_, respectively. This is visible in the differential electron density, as shown in insets of Figure 3d. The differential electron density upon adsorption confirms that CO_2_ is still physisorbed despite relatively strong binding energy.

**Figure 4:**
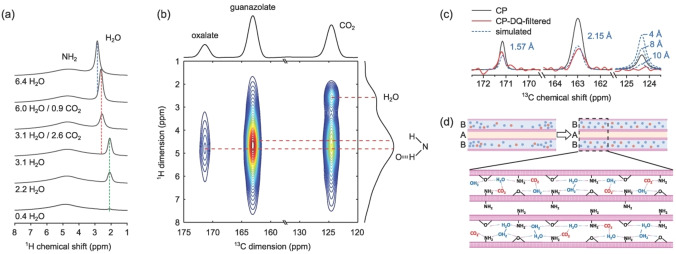
(a) ^1^H MAS NMR spectra of NICS‐24 with varying CO_2_/H_2_O compositions. Dashed lines highlight three distinct local environments of water molecules, distinguished by different numbers and/or H‐bond strengths: partially‐hydrated (green), fully‐hydrated (blue), and CO_2_‐water mixed phases (red). (b) ^1^H‐^13^C CP‐HETCOR NMR spectrum of NICS‐24 with 6 H_2_O and 0.9 CO_2_ molecules per unit cell. Dashed lines indicate ^1^H chemical shifts of water and two NH_2_ protons, with one slightly donwnfield shifted due to H‐bonding, (c) ^1^H‐^13^C CP‐MAS (black solid) and ^13^C CP‐DQ‐filtered (red solid) NMR spectra of NICS‐24 containing 6 H_2_O and 0.9 CO_2_ molecules per unit cell, alongside simulated 13 C CP‐DQ‐filtered (blue dashed) peaks for oxalates (C−C distances of 1.57 Å), guanazolates (C−C distance of 2.15 Å), and CO_2_ molecule with C−C distances of 4 Å, 8 Å and 10 Å, assuming 99 % ^13^C‐enrichment of CO_2_. (d) Schematic representation of CO_2_/H_2_O adsorption mechanism, illustrating the initial clustering of adsorbate molecules close to the entrance of B‐type channels, whereas A‐type channels remain empty (upper left), and the tendency for homogeneous distribution and formation of CO_2_‐H_2_O pairs at the equilibrium (upper right and down).

To elucidate the formation of hydrogen bonds and competitive adsorption of CO_2_ and H_2_O in NICS‐24, we employed ^1^H, ^13^C, and ^15^N MAS NMR spectroscopy. Samples were prepared ex situ with varying amounts of H_2_O and CO_2_. Quantitative analysis of the ^1^H and ^13^C MAS spectra was used to determine the precise molar amount of H_2_O/CO_2_ per unit cell. The ^1^H MAS spectra exhibit a broad signal at 4.6 ppm attributed to the protons of NH_2_ functional groups, and a narrow signal between 2–3 ppm corresponding to water protons (Figure 4a). The ^13^C MAS NMR confirms the expected 1 : 2 ratio between oxalate carbon nuclei (around 171 ppm) and guanazolate carbon nuclei (163 ppm) and reveals an additional peak at 124 ppm assigned to encapsulated CO_2_ (Figure S33). Strong spinning sidebands of the CO_2_ peak at slower MAS rates indicate restricted dynamics of CO_2_, suggesting binding within the micropores of NICS‐24. The ^1^H‐^13^C CP‐HETCOR spectrum shows a cross‐peak between CO_2_ and NH_2_ resonating at slightly shifted ^1^H frequency (4.8 ppm), indicating deshielding of the NH_2_ proton due to hydrogen bonding with CO_2_ (Figure S34). The restricted dynamics of hydrogen‐bonded NH_2_ prevent averaging of the chemical shifts between the two inequivalent protons attached to the same nitrogen atom. The unbound NH_2_ proton resonates at ~4.4 ppm and is also in close proximity to the CO_2_ carbon, suggesting that the CO_2_ molecule is slightly tilted relative to the axis of symmetry of the NH_2_ group. Surprisingly, CO_2_ also interacts with H_2_O, giving rise to another correlation peak in the ^1^H‐^13^C CP‐HETCOR at the ^1^H position of 2.6 ppm. This signal is shifted by ~0.6 ppm downfield compared to the sample with the same amount of water but without CO_2_, confirming the OCO⋅⋅⋅HO hydrogen bond formation. ^1^H‐^13^C CP‐HETCOR also supports the formation of hydrogen bond between oxalate oxygen and NH_2_ of guanazolate linker, which is consistent with the proposed structure and DFT calculations.

Amine groups are known for their hydrophilicity and the ^1^H‐^1^H SQ‐SQ homonuclear‐correlation NMR experiment indeed confirms proximities between H_2_O and NH_2_ species (Figure S35a), and ^1^H‐^1^H DQ‐SQ, which is more sensitive to shorter distances and can in addition to SQ‐SQ detect correlations between equivalent protons, further confirms the existence of two distinct proton sites in NH_2_ moieties, with one slightly shifted due to hydrogen bond formation (Figure S35b). Moreover, in ^1^H‐^15^N CPMAS spectrum we can also resolve two NH_2_ sites as evident from Figure S35b, but ^1^H‐^15^N CP‐HETCOR clearly shows these two sites are both involved in hydrogen bonding to some extent as there is hardly any difference in the center of the gravity for the two cross‐peaks along ^1^H dimension (Figure S36). PXRD analysis revealed that half of NH_2_ groups point towards B channels and the other half towards A channels, experiencing slightly different local environments, resulting in peak splitting. The rest of the ^15^N peaks of guanazolate linkers were not split but were still influenced by the content inside the large pores due to the nearby NH_2_ groups involved in host–guest hydrogen bonding. Conversely, chemical shift of NH_2_ within B channels seems less affected by guest molecules, suggesting no molecules enter the smaller channels. Similarly, the ^13^C peak position of oxalates shifted with increasing water content, further confirming interaction of oxalates with H_2_O inside the large pores (Figure S36). With solid‐state NMR we were able to follow different sorption sites. ^1^H MAS NMR showed that with up to four H_2_O molecules per unit cell, water molecules reside in magnetically equivalent local environments, forming weak NH⋅⋅⋅OH and OH⋅⋅⋅OCC hydrogen bonds, resulting in slight deshielding of the water protons from the expected 1.5 ppm for isolated water molecules, shifting to about 2 ppm in NICS‐24. When more than four H_2_O molecules per unit cell are introduced, additional or slightly stronger hydrogen bonds are formed (most likely between the neighboring H_2_O), shifting the peak to a final position of 2.85 ppm. Unlike in case of NH_2_, the diffusion of water and/or proton hopping prevents the observation of distinct water protons; however, the averaged chemical shift serves as a good indicator of the hydrogen‐bonded network strength.

In NICS‐24, the interplay between CO_2_ and H_2_O molecules is inevitable. The total number of O atoms per unit cell remains constant at 8, indicating that every molecule of CO_2_ is replaced by two water molecules after air exposure, forming a slightly stronger hydrogen‐bonded network (2.85 ppm vs. 2.6 ppm). But even when the material is partially filled with water (signal at 2 ppm), it continues to strongly adsorb either CO_2_ or preferentially H_2_O. Furthermore, CO_2_ has a tendency to evenly distribute along the channels without clustering, as no CO_2_‐CO_2_ pairs were detected at C−C distances up to 10 Å, evident from the ^13^C CP‐DQ‐filtered experiment (Figure 4c). Interestingly, this tendency of forming H_2_O‐CO_2_ pairs is stronger than preserving fully‐water occupied unit cells in the equilibrium. A phase segregation was observed initially when H_2_O was introduced into CO_2_‐loaded NICS‐24, with separate H_2_O‐H_2_O and H_2_O‐CO_2_ domains (Figure S35). After several days of the sample in a closed rotor, CO_2_ redistributed uniformly within the framework, as evidenced by a single ^1^H peak at ~2.6 ppm. This redistribution (shown in Figure 4d) illustrates the framework‘s ability to dynamically adjust to varying guest molecule ratios. After the rotor was opened and exposed to air, CO_2_ exchanged with water vapor, causing a shift of the ^1^H signal to 2.85 ppm, highlighting the material's preference for water over CO_2_ adsorption under ambient conditions.

In summary, the study revealed that the amine groups within NICS‐24 enhance CO_2_ interactions through specific hydrogen bonding, which is modulated by the presence of water. Initially, CO_2_ and water cluster near type B channel entrances and are then redistributed over time, achieving a homogenized equilibrium state characterized by specific interactions with the framework sorption sites and between each other (Figure 4d). Unlike the commonly reported amine‐functionalized MOF adsorbents, NICS‐24 does not exhibit cooperative CO_2_‐H_2_O adsorption via hydronium carbamate formation. This behaviour is attributed to its structural and chemical properties: (a) the amine groups in NICS‐24 are not spatially aligned to stabilize such species, as cooperative adsorption requires closely positioned amines for carbamate formation; (b) its strong hydrophilicity, confirmed by NMR and computational analyses, favours water competing for adsorption sites, limiting CO_2_ binding efficiency, (c) its ultra‐microporous channels impose steric constraints, preventing the formation of bulkier species. These findings emphasize the balance between competition and synergy in NICS‐24, driven by its framework properties, and highlight its capability for CO_2_ capture under humid conditions despite efficiency trade‐offs.

## Conclusion

This study establishes NICS‐24, a diaminotriazole oxalate Zn(II)‐based MOF, as a promising material for low‐concentration CO_2_ capture, particularly in indoor environments. Through the incorporation of amine functionalities, NICS‐24 exhibited significant enhancements in CO_2_ adsorption, achieving an 8‐fold increase in uptake compared to the non‐amine functionalized CALF‐20 and a 40 % improvement over the mono‐amino CALF‐15 at indoor‐relevant conditions (2000 ppm CO_2_ at 25 °C). These findings demonstrate the efficacy of tailoring framework chemistry to strengthen CO_2_‐framework interactions, enabling effective adsorption at low partial pressures.

Enhanced CO_2_ binding comes at the cost of increased hydrophilicity, leading to competitive water adsorption in humid conditions. Multi‐aspect approach which included dynamic breakthrough experiments, supported with computational DFT and experimental atomistic solid‐state NMR revealed that the strong hydrogen bonding between water molecules and the amine groups in NICS‐24 diminishes CO_2_ uptake by occupying key adsorption sites. This trade‐off between CO_2_ affinity and water resistance underscores the challenges in designing MOFs for realistic, moisture‐rich environments. Despite this limitation, the structural integrity of NICS‐24 remained intact, indicating its robustness and adaptability for further optimization.

The study highlights a critical balance that must be achieved in the design of MOFs for CO_2_ capture: while strong binding energies are desirable for capturing low‐concentration CO_2_, they can also lead to challenges in humid environments where water competes for the same adsorption sites. This trade‐off between binding energy strength and efficient CO_2_ capture in diluted, wet conditions underscores the need for fine‐tuning the material‘s properties by a proper structural modification or composite material to mitigate water interference while maintaining high CO_2_ capture performance.

The authors have cited additional references within the Supporting Information.[^24^, ^30^, ^31^, ^35^, ^45^, ^46^, ^47^, ^48^, ^49^, ^50^, ^51^, ^52^, ^53^, ^54^, ^55^, ^56^, ^57^, ^58^, ^59^, ^60^, ^61^, ^62^, ^63^, ^64^, ^65^, ^66^, ^67^, ^68^, ^69^, ^70^, ^71^, ^72^, ^73^, ^74^]

## Conflict of Interests

The authors declare no conflict of interest.

1

## Supporting information

As a service to our authors and readers, this journal provides supporting information supplied by the authors. Such materials are peer reviewed and may be re‐organized for online delivery, but are not copy‐edited or typeset. Technical support issues arising from supporting information (other than missing files) should be addressed to the authors.

Supporting Information

## Data Availability

The data that support the findings of this study are available from the corresponding author upon reasonable request.

## References

[1] K. W. Tham , Energy Build. 2016, 130, 637.

[2] S. A. Ahmed Abdul-Wahab , S. C. F. En , A. Elkamel , L. Ahmadi , K. Yetilmezsoy , Atmos. Pollut. Res. 2015, 6, 751–767.

[3] D. P. Wyon, *Indoor Air* **2004**, *14* (7), 92. 10.1111/j.1600-0668.2004.00278.x 15330777

[4] C. Kim , C.-J. Yoo , H.-S. Oh , B. K. Min , U. Lee , J. CO2 Util. 2022, 65, 102239.

[5] R. S. Norhasyima , T. M. I. Mahlia , J. CO2 Util. 2018, 26, 323.

[6] S. Valluri , V. Claremboux , S. Kawatra , J. Environ. Sci. 2022, 113, 322. 10.1016/j.jes.2021.05.043 34963541

[7] L. Jiang , W. Liu , R. Q. Wang , A. Gonzalez-Diaz , M. F. Rojas-Michaga , S. Michailos , M. Pourkashanian , X. J. Zhang , C. Font-Palma , Prog. Energy Combust. Sci. 2023, 95, 101069.

[8] F. Zerobin , T. Pröll , Ind. Eng. Chem. Res. 2020, 59, 9207.

[9] Z. Chen , K. O. Kirlikovali , L. Shi , O. K. Farha , Mater. Horiz. 2023, 10, 3257. 37285170 10.1039/d3mh00541k

[10] Z. Ji , H. Wang , S. Canossa , S. Wuttke , O. M. Yaghi , Adv. Funct. Mater. 2020, 30, 2000238.

[11] J. Canivet , A. Fateeva , Y. Guo , B. Coasne , D. Farrusseng , Chem. Soc. Rev. 2014, 43, 5594. 24875439 10.1039/c4cs00078a

[12] J. An , O. K. Farha , J. T. Hupp , E. Pohl , J. I. Yeh , N. L. Rosi , Nat. Commun. 2012, 3, 604. 22215079 10.1038/ncomms1618

[13] G. Centi , S. Perathoner , Microporous Mesoporous Mater. 2008, 107, 3.

[14] S. Mahajan , M. Lahtinen , J. Environ. Chem. Eng. 2022, 10, 108930.

[15] R. Babarao , J. Jiang , Langmuir 2008, 24, 6270. 18484751 10.1021/la800369s

[16] Q. Yang , C. Zhong , J. F. Chen , J. Phys. Chem. C 2008, 112, 1562.

[17] A. Torrisi , R. G. Bell , C. Mellot-Draznieks , Cryst. Growth Des. 2010, 10, 2839.

[18] C. A. Trickett , A. Helal , B. A. Al-Maythalony , Z. H. Yamani , K. E. Cordova , O. M. Yaghi , Nat. Rev. Mater. 2017, 2 (8), 17045.

[19] A. Masala , J. G. Vitillo , G. Mondino , C. A. Grande , R. Blom , M. Manzoli , M. Marshall , S. Bordiga , ACS Appl. Mater. Interfaces 2017, 9 (1), 455–463. 28005324 10.1021/acsami.6b13216

[20] A. Koutsianos , E. Kazimierska , A. R. Barron , M. Taddei , E. A. Andreoli , Dalton Trans. 2019, 48, 3349. 30778497 10.1039/c9dt00154a

[21] O. Shekhah , Y. Belmabkhout , Z. Chen , V. Guillerm , A. Cairns , K. Adil , M. Eddaoudi , Nat.Comm. 2014, 5, 4228. 10.1038/ncomms5228 PMC4083436 24964404

[22] P. M. Bhatt , Y. Belmabkhout , A. Cadiau , K. Adil , O. Shekhah , A. Shkurenko , L. J. Barbour , M. Eddaoudi , J. Am. Chem. Soc. 2016, 138 (29), 9301–9307. 27388208 10.1021/jacs.6b05345

[23] Y. Hu , Y. Jiang , J. Li , L. Wang , M. Steiner , R. F. Neumann , B. Luan , Y. Zhang , Adv. Funct. Mater. 2023, 33, 2213915.

[24] T. M. McDonald , W. R. Lee , J. A. Mason , B. M. Wiers , C. Seop Hong , J. R. Long , J. Am. Chem. Soc. 2012, 134, 7056–7065. 22475173 10.1021/ja300034j

[25] J. M. Kolle , M. Fayaz , A. Sayari , Chem. Rev. 2021, 121, 7280. 33974800 10.1021/acs.chemrev.0c00762

[26] W. R. Lee , S. Y. Hwang , D. W. Ryu , K. S. Lim , S. S. Han , D. Moon , J. Choi , C. S. Hong , Energy Environ. Sci. 2014, 7, 744.

[27] H. Dong , L.-H. Li , Z. Feng , Q.-N. Wang , P. Luan , J. Li , C. Li , ACS Materials Lett. 2023, 5, 2656.

[28] R. Ben Said , J. M. Kolle , K. Essalah , B. Tangour , A. Sayari , ACS Omega 2020, 5, 26125. 33073140 10.1021/acsomega.0c03727 PMC7557993

[29] D. D. Miller , J. Yu , S. S. C. Chuang , J. Phys. Chem. C 2020, 124, 24677.

[30] J.-B. Lin , T. T. T. Nguyen , R. Vaidhyanathan , J. Burner , J. M. Taylor , H. Durekova , F. Akhtar , R. K. Mah , O. Ghaffari-Nik , S. Marx , N. Fylstra , S. S. Iremonger , K. W. Dawson , P. Sarkar , P. Hovington , A. Rajendran , T. K. Woo , G. K. H. Shimizu , Science 2021, 374, 1464. 34914501 10.1126/science.abi7281

[31] R. Vaidhyanathan , S. S. Iremonger , K. W. Dawson , G. K. H. Shimizu , Chem. Commun. 2009, 35, 5230. 10.1039/b911481e 19707629

[32] R. Vaidhyanathan , S. S. Iremonger , G. K. H. Shimizu , P. G. Boyd , S. Alavi , T. K. Woo , Science 2010, 330, 650. 21030651 10.1126/science.1194237

[33] K. C. Kim , T.-U. Yoon , Y.-S. Bae , Microporous Mesoporous Mater. 2016, 224, 294–301.

[34] Y. Hu , Y. Chen , W. Yang , J. Hu , X. Li , L. Wang , Y. Zhang , Sep. Purif. Technol. 2024, 343, 127099.

[35] A. Banerjee , S. Nandi , P. Nasa , R. Vaidhyanathan , Chem. Commun. 2016, 52 (9), 1851. 10.1039/c5cc08172f 26671348

[36] Z. Hu , Y. Wang , B. B. Shah , D. Zhao , Adv. Sustainable Syst. 2018, 3, 1800080.

[37] J. A. Garcia , M. Villen-Guzman , J. M. Rodriguez-Maroto , J. M. Paz-Garcia , J. Environ. Chem. Eng. 2022, 10, 108470.

[38] F. Raganati , F. Miccio , P. Ammendola , Energy Fuels 2021, 35, 12845.

[39] L. R. López , P. Dessì , A. Cabrera-Codony , L. Rocha-Melogno , N. J. R. Kraakman , M. D. Balaguer , S. Puig , Clean. Eng. Technol. 2024, 20, 100746. 10.1016/j.scitotenv.2022.159088 36181799

[40] Y. Belmabkhout , V. Guillerm , M. Eddaoudi , Chem. Eng. J. 2016, 296, 386.

[41] A. Nuhnen , C. Janiak , Dalton Trans. 2020, 49, 10295. 32661527 10.1039/d0dt01784a

[42] K. S. Walton , D. S. Sholl , AIChE J. 2015, 61, 2757.

[43] K. Gopalsamy , D. Fan , S. Naskar , Y. Magnin , G. Maurin , ACS Appl. Energ. Mater. 2024, 2, 96.

[44] E. González-Zamora , I. A. Ibarra , Mater. Chem. Front. 2017, 1, 1471.

[45] B. Grünberg , T. Emmler , E. Gedat , I. Shenderovich , G. H. Findenegg , H. Limbach , G. Buntkowsky , Chem. A Eur. J. 2024, 10, 5689. 10.1002/chem.200400351 15470692

[46] C. Dalvit , M. Veronesi , A. Vulpetti , J. Magn. Reson. Open 2022, 12–13, 100070.

[47] M. N. C. Zarycz , C. Fonseca Guerra , J. Phys. Chem. Lett. 2018, 9, 3720. 29927254 10.1021/acs.jpclett.8b01502 PMC6038099

[48] J. Peng , H. Wang , D. H. Olson , Z. Li , J. Li , Chem. Commun. 2017, 53, 9332. 10.1039/c7cc03529b 28783197

[49] R. Zhang , Y. Nishiyama , P. Sun , A. Ramamoorthy , J. Magn. Reson. 2015, 252, 55. 25655451 10.1016/j.jmr.2014.12.010 PMC4380770

[50] M. Feike , D. E. Demco , R. Graf , J. Gottwald , S. Hafner , H. W. Spiess , J. Magn. Reson. Ser. A 1996, 122, 214.

[51] A. Altomare , C. Cuocci , C. Giacovazzo , A. Moliterni , R. Rizzi , N. Corriero , A. Falcicchio , J. Appl. Crystallogr. 2013, 46, 1231.

[52] Bruker AXS (2018) TOPAS, v 6.0.

[53] G. Metz , X. L. Wu , S. O. Smith , J. Magn. Reson. Ser. A 1994, 110, 219.

[54] A. E. Bennett , C. M. Rienstra , M. Auger , K. V. Lakshmi , R. G. Griffin , J. Chem. Phys. 1995, 103, 6951.

[55] I. Scholz , P. Hodgkinson , B. H. Meier , M. Ernst , J. Chem. Phys. 2009, 130, 114510. 19317548 10.1063/1.3086936

[56] M. Kotecha , N. P. Wickramasinghe , Y. Ishii , Magn. Reson. Chem. 2007, 45, S221. 18157841 10.1002/mrc.2151

[57] M. Hohwy , H. J. Jakobsen , M. E. Edén , M. H. Levitt , N. C. Nielsen , J. Chem. Phys. 1998, 108, 2686.

[58] M. Bak , J. T. Rasmussen , N. C. Nielsen , J. Magn. Reson. 2000, 147, 296. 11097821 10.1006/jmre.2000.2179

[59] A. E. Bennett , C. M. Rienstra , J. M. Griffiths , W. Zhen , P. T. Lansbury , R. G. Griffin , J. Chem. Phys. 1998, 108, 9463.

[60] M. Shen , B. Hu , O. Lafon , J. Trébosc , Q. Chen , J.-P. Amoureux , J. Magn. Reson. 2012, 223, 107. 22985981 10.1016/j.jmr.2012.07.013

[61] S. Choi , T. Watanabe , T. H. Bae , D. S. Sholl , C. W. Jones , J. Phys. Chem. Lett. 2012, 3, 1136–1141. 26288048 10.1021/jz300328j

[62] W. R. Lee , S. Y. Hwang , D. W. Ryu , K. S. Lim , S. S. Han , D. Moon , J. Choi , C. S. Hong , Energy Environ. Sci. 2014, 7, 744–751.

[63] H. Jo , W. R. Lee , N. W. Kim , H. Jung , K. S. Lim , J. E. Kim , D. W. Kang , H. Lee , V. Hiremath , J. G. Seo , H. Jin , D. Moon , S. S. Han , C. S. Hong , ChemSusChem 2017, 10, 541–550. 28004886 10.1002/cssc.201601203

[64] W. R. Lee , J. E. Kim , S. J. Lee , M. Kang , D. W. Kang , H. Y. Lee , V. Hiremath , J. G. Seo , H. Jin , D. Moon , M. Cho , Y. Jung , C. S. Hong , ChemSusChem 2018, 11, 1694–1707. 29603670 10.1002/cssc.201800363

[65] J. Park , J. R. Park , J. H. Choe , S. Kim , M. Kang , D. W. Kang , J. Y. Kim , Y. W. Jeong , C. S. Hong , ACS Appl. Mater. Interfaces 2020, 12, 50534–50540. 33131271 10.1021/acsami.0c16224

[66] L. A. Darunte , A. D. Oetomo , K. S. Walton , D. S. Sholl , C. W. Jones , ACS Sustainable Chem. Eng. 2016, 4, 5761–5768.

[67] H. Li , K. Wang , D. Feng , Y. P. Chen , W. Verdegaal , H. C. Zhou , ChemSusChem 2016, 9, 2832–2840. 27584839 10.1002/cssc.201600768

[68] D. Jo , S. K. Lee , K. H. Cho , J. W. Yoon , U. H. Lee , ACS Appl. Mater. Interfaces 2022, 14, 56707–56714. 36516324 10.1021/acsami.2c15476

[69] Z. Shi , Y. Tao , J. Wu , C. Zhang , H. He , L. Long , Y. Lee , T. Li , Y. B. Zhang , J. Am. Chem. Soc. 2020, 142, 2750–2754. 31968944 10.1021/jacs.9b12879

[70] O. T. Qazvini , S. G. Telfer , ACS Appl. Mater. Interfaces 2021, 13, 12141–12148. 33661605 10.1021/acsami.1c01156

[71] O. T. Qazvini , S. G. Telfer , J. Mater. Chem. A 2020, 8, 12028–12034.

[72] J. An , S. J. Geib , N. L. Rosi , J. Am. Chem. Soc. 2010, 132, 38–39. 20000664 10.1021/ja909169x

[73] S. Couck , J. F. M. Denayer , G. V. Baron , T. Rémy , J. Gascon , F. Kapteijn , J. Am. Chem. Soc. 2009, 131, 6326–6327. 19374416 10.1021/ja900555r

[74] G. E. Cmarik , M. Kim , S. M. Cohen , K. S. Walton , Langmuir 2012, 28, 15606–15613. 23057691 10.1021/la3035352

