# Supplementary material for: Amine‐Functionalized Triazolate‐Based Metal–Organic Frameworks for Enhanced Diluted CO2 Capture Performance
Source: Angew Chem Int Ed Engl. 2025 Jan 31;64(14):e202424747. doi: 10.1002/anie.202424747 (PMC11966689; doi:10.1002/anie.202424747)
Supplement: Supplementary file 1 — Supporting Information [file ANIE-64-e202424747-s001.pdf]

Angewandte Chemie
Eine Zeitschrift der Gesellschaft Deutscher Chemiker

Supporting Information

Amine-Functionalized Triazolate-Based Metal–Organic Frameworks for Enhanced Diluted CO_2 Capture Performance

K. Klemenčič, A. Krajnc, A. Puškarić, M. Huš, D. Marinič, B. Likozar, N. Z. Logar, M. Mazaj**

Supporting information

Amine-functionalized Triazolate-based Metal–Organic Frameworks for Enhanced Diluted CO2 Capture Performance

Klara Klemenčič,[a,b] Andraž Krajnc,*[a] Andreas Puškarić,[a,c] Matej Huš, [a,d,e] Dana Marinič,[a,f] Blaž Likozar,[a] Nataša Zabukovec Logar,[a,b], Matjaž Mazaj*[a]

[a] K. Klemenčič, Dr. A. Krajnc, Dr. A. Puškarić, Dr. M. Huš, Dana Marinič, Dr. B. Likozar, Dr. N. Zabukovec Logar, Dr. M. Mazaj
National Institute of Chemistry
Hajdrihova 19, 1000 Ljubljana, Slovenia
E-mail: matjaz.mazaj@ki.si, andraz.krajnc@ki.si

[b] K. Klemenčič, Dr. N. Zabukovec Logar
University of Nova Gorica
Vipavska cesta 13, 5000 Nova Gorica, Slovenia

[c] Dr. A. Puškarić
Rudjer Bošković Institute
Bijenička cesta 54, 10000 Zagreb, Croatia

[d] Dr. M. Huš
Association for Technical Culture of Slovenia (ZOTKS)
Zaloška 65, 1000 Ljubljana, Slovenia

[e] Dr. M. Huš
Institute for the Protection of Cultural Heritage (ZVKDS)
Poljanska 40, 1000 Ljubljana, Slovenia

[f] D. Marinič
Faculty of Chemistry and Chemical Engineering
University of Maribor
Smetanova 17
2000 Maribor, Slovenia

1. Synthesis

NICS-24 (Zn-3,5-diamine-1,2,4-triazolate oxalate) was synthesized using three methods:

Solvothermal synthesis with zinc oxalate dihydrate (**NICS-24-SOL-Ox**)
Synthesis was carried out by stirring 0.49 g (2.56 mmol) of zinc oxalate dihydrate (99% Aaron Chem) and 0.76 g (7.68 mmol) of guanazole (98% Aaron Chem) in 5 ml of demineralized water for 5 min. Reaction mixture was then transferred to 23 ml Parr stainless steel Teflon-lined autoclave and heated in a convection oven at 180 °C for 48 h. The product in the form of white powder containing micron-sized elongated prismatic crystals (Figure S2), was recovered by filtration, rinsed with ethanol and dried at ambient conditions. The synthesis yield was 60 % in respect to Zn(II)-precursor.

Solvothermal synthesis with zinc oxide (**NICS-24-SOL-ZnO**)
Synthesis was carried out by stirring 0.20 g (2.46 mmol) of zinc oxide (99% Aaron Chem) in 10 ml of demineralized water. Then 0.16 g (1.27 mmol) of oxalic acid dihydrate (99% Merck) and 0.61 g (6.16 mmol) of guanazole (98% Aaron Chem) were added to the solution, followed by stirring for 15 minutes at room temperature. The resulting mixture was then transferred to a 23 ml Parr stainless steel Teflon-lined autoclave and heated in a convection oven at 150 °C for 72 h. The obtained white powder was washed with ethanol and then dried at ambient conditions. The synthesis yield was 65 % in respect to Zn(II)-precursor.

Solvent-assisted ligand exchange (**NICS-24-SALE**)
Initially, 0.16 g (0.82 mmol) of prepared CALF-20 powder was dispersed in 3.5 ml of methanol (100 % Fluka) and 3.5 ml od demineralized water by stirring at room temperature for 15 minutes. Then 0.13 g (1.31 mmol) of guanazole (98% Aaron Chem) was added to the mixture. After an additional 15 minutes of stirring, the mixture was then transferred to a 23 ml Parr stainless steel Teflon-lined autoclave and heated in a convection oven at 120 °C for 72 hours. The obtained white powder was washed with ethanol and then dried at ambient conditions. The synthesis yield was 81 % in respect to CALF-20.

CALF-20 (Zn-1,2,4-triazolate oxalate) reference. Synthesis of CALF-20 was performed according to the published data.[1] Typically, mixture of 1.32 g (6.97 mmol) of zinc oxalate dihydrate (99% Aaron Chem), 1.00 g (14.48 mmol) of 1,2,4-triazole (97 % Fluorochem) and 13 ml of methanol (100%, Fluka) was heated in 23 ml Parr stainless steel Teflon-lined autoclave at 180 °C for 48 h. The product in the form of white powder was recovered by filtration, rinsed with ethanol and dried at ambient conditions. Adequacy of the product was confirmed by powder XRD measurement (Figure S6).

CALF-15 (Zn-3-amine-1,2,4-triazolate oxalate) reference. Synthesis of CALF-15 was performed according to the published data.[2] Typically, mixture of 0.66 g (3.48 mmol) of zinc oxalate dihydrate (99% Aaron Chem), 1.46 g (17.36 mmol) of 3-amine-1,2,4-triazole (Sigma Aldrich) and 3.5 ml of butanol (99.9% Sigma Aldrich) and 3.5 ml of demineralized water was heated in 23 ml Parr stainless steel Teflon-lined autoclave at 180 °C for 72 h. The product in the form of white powder was recovered by filtration, rinsed with ethanol and dried at ambient conditions. Adequacy of the product was confirmed by powder XRD measurement (Figure S6).

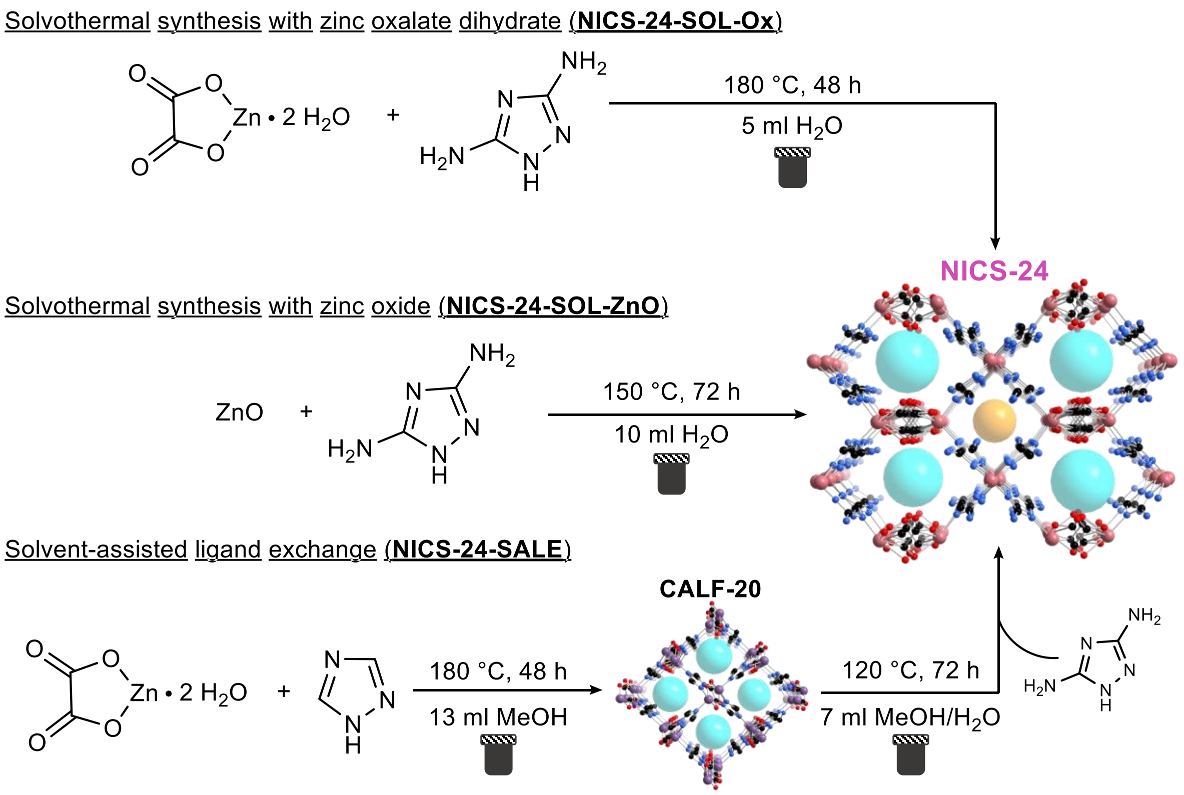

Figure S1. Scheme indicating used methods for the synthesis of NICS-24.

2. Characterization methods

Powder XRD data were collected on a PANalytical X'Pert PRO diffractometer using CuKα radiation (λ = 1.5418 Å) at room temperature in an angular range of 5 – 50° 2θ with a step size of 0.034° and 100 s per step using fully-opened 100 channel X'Celerator detector. Morphology, phase purity and size of the crystals were observed by Zeiss Supra™ 3VP fieldemission gun (FEG) scanning electron microscope. Thermogravimetric measurements were performed on a TA Instruments Q5000 apparatus in air flow of 10 ml/min at the heating rate of 5 °C/min. Sorption data for CO_2 and N_2 were collected on IQ3 Anton Paar adsorber. Prior the measurements typically 30 mg of samples were outgassed at 60 °C for 2h and 100 °C for 10 h. Specific surface area was determined by BET theory based on CO_2 isothermal data measured at 273 K (Figure S11) in the relative pressure region between 0.00027 – 0.0015 resulting in highest linearity correlation (Figure S12). Pore size distribution was determined by NLDFT using a model, which included CO_2 as adsorbate and carbon as adsorbent at 273K (Figure S13). Isosteric heat of adsorption calculations were based on Clausius-Clapeyron method using isothermal data measured at 273 K, 283 K and 293 K. Isotherms were fitted using Dual site Langmuir model (Equation 1, Figures S14-S16, Table S6):

$$q_e = \frac{q_{m1}K_1P}{1+K_1P} + \frac{q_{m2}K_2P}{1+K_2P}, \qquad \text{Eq1}$$

where q_e is the equilibrium adsorbed amount at corresponding partial pressure P, q_{m1} and q_{m2} are maximum adsorption capacities for sites 1 and 2 respectively, K_1 *and* K_2 are Langmuir constants for sites 1 and 2 respectively.

Water isotherms were performed on IGASorp dynamic vapor sorption analyzer (Hiden Isochema) at 25 °C in the range of 10 – 90 %RH using a flow of 10 ml/min with N_2 carrier gas. CO_2 sorption cycling experiments were realized in DVS Vacuum gravimetric analyser (Surface Measurements System) in static mode dosing CO_2 to 1 bar from vacuum. Prior the measurements, samples were outgassed using identical protocols - 60 °C for 2h and 100 °C for 10 h.

Breakthrough measurements were conducted on the adsorbents by using the Catalyst Analyzer Belcat II (Microtrac, Montgomeryville and York, Pennsylvania, USA). Approximately 50 mg of sorbent was loaded in the column with the length of 18 cm and diameter of 1cm, with the packed bad having of 0.5 cm in height. The column operates under constant atmosphere pressure conditions. The adsorbents were regenerated in pure He (50 ml/min) at 60 °C for 2 hours and at 120 °C for 10 hours at the heating rate of 10 °C/min for the purpose of desorbing any pre-adsorbed H_2O and CO_2. The cleaning step was followed by an adsorption process at 25°C, during which a He gas mixture containing CO_2 (2000 ppm) and O_2 (20%) was fed into the adsorber column at a flow rate of 50 ml/min until the sorbent saturation. Additionally, experiments were conducted in simulated air conditions at 25°C and 50% RH (relative humidity). The total flow rate was consistently maintained at 50 ml/min, with the individual gas flow rates adjusted to achieve the desired gas composition. The desorption was performed in a helium flow of 50 ml/min at 120 °C until the sorbent was fully regenerated. The gases exiting the reactor were analyzed using mass spectrometry (MS), providing a mass chromatogram that plots signal intensity versus time for specific ions. Calibration was performed to determine the amount of gas at the outlet over time. The MS scan speed was set to 1 s per atomic mass unit, ensuring continuous data collection. Temperature and pressure were also measured throughout the experiments. The analysis focused on determining the saturation time, the amount of CO_2 and H_2O adsorbed, and the impact of water on the kinetics of CO_2 adsorption, and vice versa. Dead volume was measured through a blank experiment (using a sample cell filled with quartz wool but no adsorbent). The breakthrough curve from the blank experiment was then subtracted from the breakthrough curve of the actual measurement, effectively eliminating the contribution of the dead volume from the results. The data also provided the breakthrough time, which marked the curve integration starting point. The breakthrough curves were integrated to calculate the amount of adsorbed gas, which was then normalized by the mass of the sorbent to determine the adsorption capacity.

All NMR experiments were conducted on a 600 MHz Varian NMR system equipped with a 1.6 mm FastMAS Varian probe. The two-dimensional (2D) spectra were recorded at a magicangle spinning (MAS) frequency of 20 kHz, while the one-dimensional spectra were recorded at 25 kHz, unless otherwise specified. The Larmor frequencies for ^{1}H, ^{13}C, and ^{15}N nuclei were 599.7 MHz, 150.8 MHz, and 60.8 MHz, respectively. Frequency axes for ^{1}H and ^{13}C spectra were referenced to tetramethylsilane, while those for ^{15}N were referenced relative to nitromethane. For the ^{1}H MAS, ^{13}C MAS, and ^{1}H-^{15}N cross-polarization (CP)-MAS NMR measurements, the numbers of scans were 64, 256, and 35,000, respectively, with repetition delays of 10 s, 60 s, and 1.7 s, respectively. A Hahn-echo pulse sequence was employed for the ^{1}H and ^{13}C MAS

measurements, utilizing ^{1}H π/2 pulse of 1.5 μs and 13C π/2 pulse of 1.6 μs, with π pulses set to twice these durations. An echo delay of 40 μs was applied between the π/2 and π pulses in both experiments. Additionally, a ^{13}C MAS spectrum was recorded at a slower spinning rate of 8 kHz to analyze the sideband pattern of the CO_2 signal; a single-pulse excitation sequence was employed with 1200 scans and a repetition delay of 1 s. The ^{1}H- ^{15}N CP-MAS experiment utilized a ramped amplitude[3] during the 4.0 ms CP block, followed by high-power XiX heteronuclear decoupling[4-6] during acquisition. This experiment was performed at sample spinning frequency of 20 kHz. The ^{1}H-^{13}C CP-MAS spectrum was acquired with a repetition delay of 1.7 s, 16,000 scans, and a 5 ms ramped amplitude CP block. The ^{13}C CP-double quantum (DQ)-filtered experiment commenced with a ^{1}H-^{13}C CP block and included 64 POST-C7 blocks (8 ms)[7] for the excitation of ^{13}C DQ coherences, followed by another 64 POST-C7 blocks for the reconversion of DQ coherences to zeroquantum coherences. A total of 34,000 scans were accumulated with a repetition delay of 1.7 s and sample spinning frequency of 16 kHz. The expected intensities of the DQ-filtered signals for isolated C-C spin pairs at varying distances were calculated using the SIMPSON software package[8]. Appropriate weighting factors were applied to account for the different probabilities of carbon sites being occupied by NMR-active ^{13}C nuclei in linkers and ^{13}Clabelled CO_2. The 2D ^{1}H-^{15}N CP-HETCOR spectrum was an extended CP-MAS experiment comprising 16 increments along the indirectly detected dimension with a spectral width of 10 kHz. Each increment was recorded with a 3 s delay and 2600 scans. For the 2D ^{1}H-^{13}C CPHETCOR spectrum, 20 increments were recorded with a spectral width of 10 kHz in the indirect dimension, accumulating 1024 scans per increment with a repetition delay of 1.8 s. The 2D ^{1}H-^{1}H single-quantum (SQ)-SQ homonuclear correlation experiment consisted of 60 increments with a spectral width of 20 kHz, 16 scans per increment, a repetition delay of 1 s, and a spin-diffusion mixing time of 2 ms. During the mixing period, radio-frequency driven recoupling (RFDR)[9-11] was employed. The 2D 1H-1H DQ-SQ homonuclear correlation spectrum was obtained using the back-to-back recoupling sequence (DQ-BABA)[12]. Doublequantum coherence excitation and reconversion were achieved using a single BABA cycle, with a 50 μs delay preceding the π/2 read-out pulse of 1.5 μs. The spectral width in the indirect dimension was 20 kHz, with 100 increments recorded, each with a 1 s repetition delay and 16 scans.

Structure determination of NICS-24. Crystal structure of NICS-24 (Zn-oxalate 3,5-diamino1,2,4-triazolate) was solved based on a XRD powder data obtained from PANalytical X'Pert PRO diffractometer CuKα radiation (λ = 1.5418 Å) at room temperature in an angular range of 5 – 90° 2θ with a step size of 0.034° per 300 s using fully-opened 100 channel X'Celerator detector. Unit cell parameters and space group were first determined with NTREOR algorithm. Initial model was determined ab-initio by direct methods using EXPO2014 software package.[13] Approximate positions of all atoms were successfully extracted from the obtained Patterson electron density map. Atom assignment and position corrections were done manually according to geometrical expectancies for the involved ligands. The obtained model was applied for the Rietveld refinement procedure using Topas Academic v.6 software package.[14] XRD pattern was first refined by Le Bail fit including zero error, scale fit, background fit using 9th order of Chebyshev polynomial function, pseudoVoigt peak shape fit and Suortti-Pitschke intensity corrections. Whole Rietveld analysis included gradual refinement of the unit cell parameters, isotropic displacement parameters, atom positions with the employed geometrical restrains on the ligand moieties and at the end spherical harmonics preferential orientation corrections. Final refinement included 122 independent parameters resulting in

satisfactory agreement between model and experimental data (Figure S5, Table S1). Asymmetric unit, atom position, selected interatomic distances and angles are shown in Figure S3 and Tables S2-S4.

Computational methods. The structure of NICS-24 and its adsorption properties were elucidated using the density functional theory (DFT) calculations. Using the plane-wave formalism, they were performed in VASP 6.3.1. The electron-nucleus interactions were described with the projected-augmented wave (PAW) formalism and the exchange correlation energy was modelled within the generalized gradient approximation with the PBE functional. For the van der Waals interactions, a Grimme D3 correction was employed. For well converged results, an energy cut-off of 500 eV was sufficient. The unit cell was sampled with 4x4x4 Monkhorst-Pack k-points. A Gaussian smearing of 0.03 was used to improve convergence without perturbing the results.

After optimizing the unit cell size and atomic positions, molecules of CO_2 or H_2O were introduced in the system. After finding their optimal positions, the adsorption interactions was calculated as

$$E_{ads} = E_{NICS-24+adsorbate} - E_{NICS-24} - E_{adsorbate},$$

where $E_{NICS-24+adsorbate}$ is the full energy of the system, $E_{NICS-24}$ is the energy of optimized NICS-24 structure and $E_{adsorbate}$ is the (gamma-point-sampled) energy of CO_2 or H_2O in vacuum. The Gibbs free energy of adsorption is calculated in the harmonic approximation, where only vibrational degrees of freedom are considered for adsorbates. Gaseous species are approximated as ideal gases.

The upper limit of material capacity is determined by the maximum number of adsorbate molecules, *n*, where the differential adsorption energy, defined as $E_{ads}(n) - E_{ads}(n-1)$ remains negative. The capacity (or gas uptake) is defined as

$$\text{gas uptake} = \frac{n}{\rho V_{\text{molar}}}$$

Where ρ is the (calculated) material density and V_{molar} is the molar volume of the unit cell.

Monte Carlo simulations with 20.000 hits were carried out on the optimized structure with Zeo++. Using the Voronoi decompositions, the diameter of micropore channels, the accessible channel volume, largest included sphere diameter and surface area were calculated.

The model of NICS-24 was subjected to structure relaxation using DFT for the purposes computational analysis of preferential binding sites of CO_2 described further on in the text. Theoretical simulations yield the unit cell constants for NICS-24 of a = 8.38 Å, b = 24.17 Å and c = 7.07 Å and β = 103.65°, yielding V = 1434.76 Å^3, which is within 1.3 % of the experimentally determined value. With four formula units per unit cell (1659.89 g/mol), the theoretically calculated density is thus 1.92 g/cm^3 (compare Table S1 for experimentally determined values). The computationally determined diameter of micropore channels is thus 3.8 and 5.0 Å, which is consistent with experimental data. The diameter of the largest included sphere is 3.88 Å,

which is consistent with the observed pore size distribution with the peak at 0.39 nm. The surface area is calculated as 185.5 m^2/cm^3 and accessible channel volume as 860.8 $Å^3$ when using a probe with a radius of 1.5 Å (roughly corresponding to CO_2 kinetic dimensions).

3. Structural properties

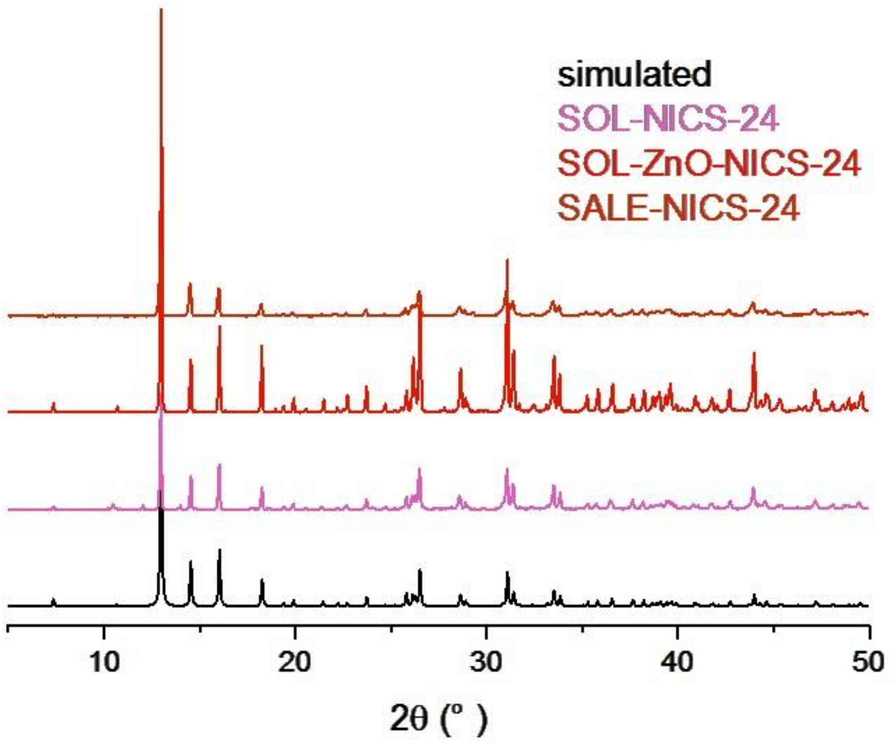

Figure S2. XRD powder patterns of NICS-24 products synthesized using specified procedures.

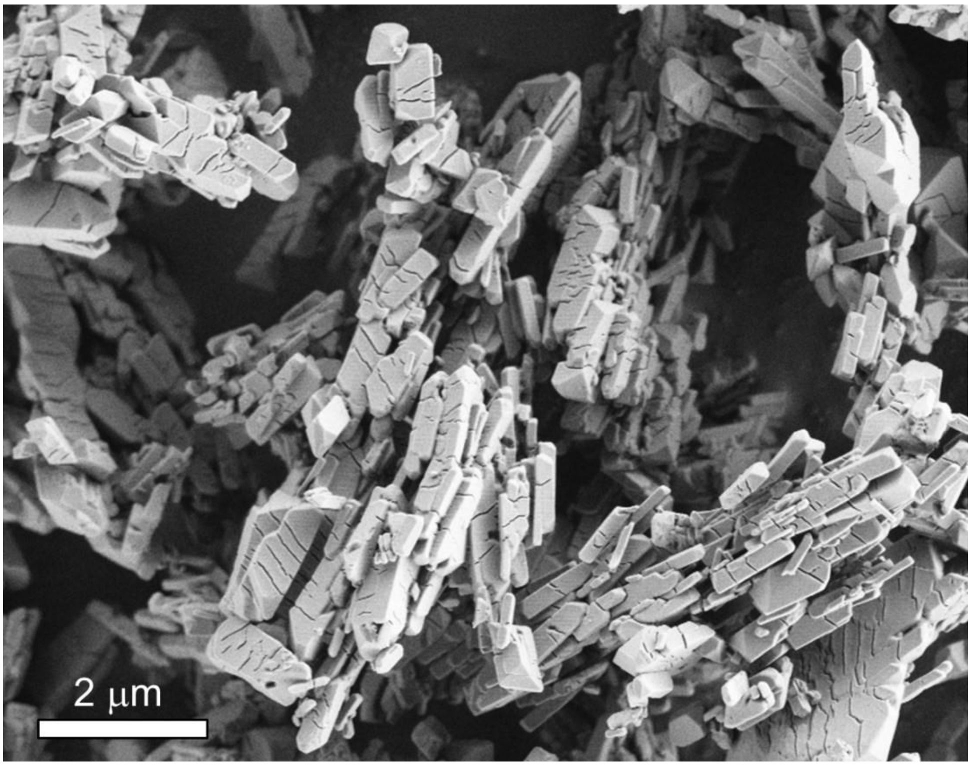

Figure S3. SEM micrograph of NICS-24 product with elongated prismatic crystals with the typical size of 0.2 x 0.2 x 1.5 μm.

Table S1. Crystal data and structure refinement parameters for NICS-24.

	**NICS-24**
CCDC	2380373
Empirical formula	$C_6H_8N_{10}O_4Zn_2$
M_w	415.0
Colour/Shape	white prismatic
Crystal system	monoclinic
Space group	$P2_1/c$
a (Å)	8.5703(3)
b (Å)	23.9768(9)
c (Å)	7.3145(3)
β (°)	104.892(2)
V (Å^3)	1452.6(1)
Z	12
T (K)	293
Wavelength (Å)	1.5406
D_{calc} (g/cm$_3$)	2.0069
R_{wp}(all data)	10.2
R_p	7.6
GOF	3.2

Table S2. Atomic positions and isotropic displacement parameters of NICS-24 structure (hydrogen atoms are excluded from the refinement).

atom	x	y	z	B_{iso} (Å^2)
Zn1	0.496(2)	0.1653(2)	0.1653(2)	0.032(2)
Zn2	0.997(2)	-0.0116(1)	0.249(29	0.032(2)
N1	0.318(6)	0.107(2)	0.165(7)	0.030(3)
N2	0.139(6)	0.038(2)	0.133(6)	0.030(3)
N3	0.121(6)	0.062(2)	-0.039(7)	0.030(3)
N4	0.242(4)	0.136(2)	-0.163(5)	0.030(3)
N5	0.314(4)	0.054(1)	0.447(6)	0.030(3)
N6	0.873(6)	0.053(2)	0.527(7)	0.030(3)
N7	0.842(6)	0.038(2)	0.339(6)	0.030(3)
N8	0.691(6)	0.115(2)	0.346(7)	0.030(3)
N9	0.771(4)	0.130(2)	0.702(5)	0.030(3)
N10	0.673(4)	0.074(1)	0.042(6)	0.030(3)
O1	0.413(5)	0.319(2)	0.005(6)	0.043(3)
O2	0.576(5)	0.183(2)	0.011(6)	0.043(3)
O3	0.335(6)	0.240(2)	0.124(7)	0.043(3)
O4	0.659(6)	0.263(2)	-0.115(7)	0.043(3)
C1	0.566(7)	0.233(2)	-0.03(1)	0.029(4)
C2	0.424(8)	0.268(2)	0.04(1)	0.029(4)
C3	0.259(7)	0.066(2)	0.25(1)	0.029(4)
C4	0.230(8)	0.103(2)	-0.02(1)	0.029(4)
C5	0.731(7)	0.076(2)	0.23(1)	0.029(4)
C6	0.782(8)	0.099(3)	0.53(1)	0.029(4)

Table S3. Selected interatomic distances in NICS-24 structure.

Bond type	Bond distances (Å)
Zn1-N8	2.04(5)
Zn1-N1	2.04(4)
Zn1-O2	2.10(5)
Zn1-O1	2.13(5)
Zn2-N6	1.99(4)
Zn2-N3	2.00(4)
Zn2-N7	2.02(5)
N1-C4	1.35(8)
N1-C3	1.35(8)
N2-C3	1.35(7)
N3-C4	1.35(7)
N4-C4	1.35(8)
N5-C3	1.40(8)
N6-C6	1.35(8)
N2-C3	1.38(8)
N3-C7	1.38(8)
N7-N6	1.38(6)
N7-C5	1.40(7)
N8-C5	1.35(8)
N8-C6	1.40(8)
N9-C6	1.49(8)
N10-C5	1.35(8)
O1-C2	1.27(6)
O2-C1	1.23(6)
O3-C2	1.28(9)
O4-C1	1.35(9)

Table S4. Selected angles between atoms in NICS-24 structure.

Atom 1	Atom 2	Atom 3	Angle 1,2,3 (°)
C4	N1	N4	127(6)
C4	N3	N4	122(5)
C4	N3	N2	106(4)
N3	N2	C3	107(5)
N2	C3	N5	125(6)
N5	C3	N1	124(5)
C3	N1	C4	103(5)
N8	C5	N10	125(5)
N10	C5	N7	125(5)
C5	N7	N6	108(4)
N7	N6	C6	104(4)
N6	C6	N9	123(6)
N9	C6	N8	123(5)
C6	N8	C5	103(5)
O2	C1	O4	127(5)
O1	C2	O3	127(5)

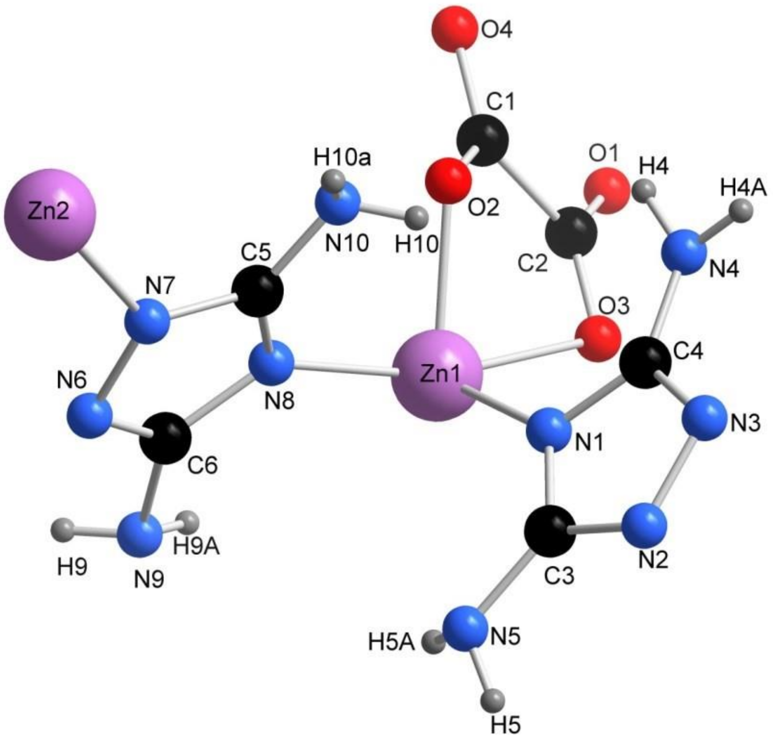

Figure S4. Asymmetric unit for NICS-24 structure. Purple balls – Zn atoms, black balls – C atoms, blue balls – N atoms, grey balls – H atoms.

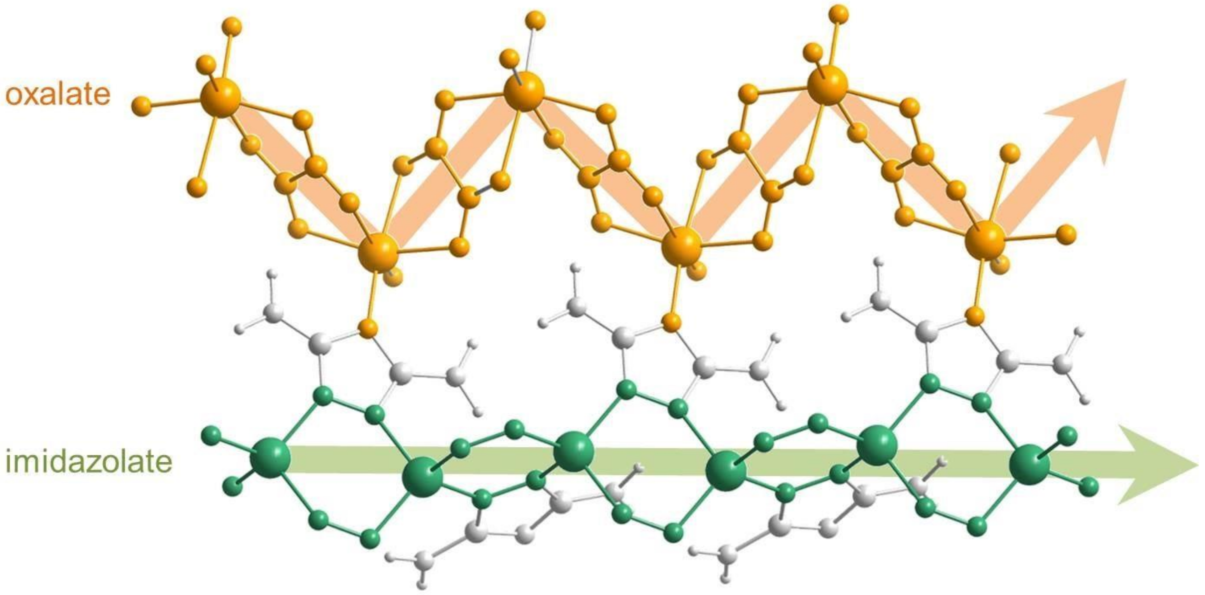

Figure S5. Scheme of the NICS-24 structure indicating two type of Zn-centered chains – zigzag oxalate-based (ocre) and straight imidazolate-based (green) chain. Atoms which are not involved in Zn(II) connectivity are coloured in light grey.

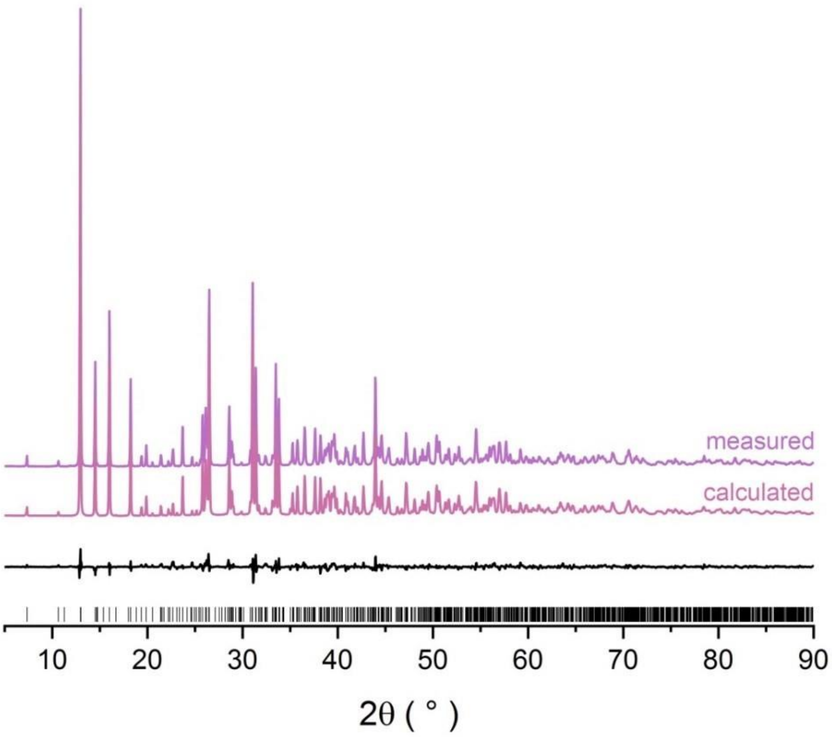

Figure S6. Rietveld plot for NICS-24 structure with the comparison of measured and calculated XRD patterns (upper and lower plots respectively), difference plot (black line) and calculated peak positions (black ticks).

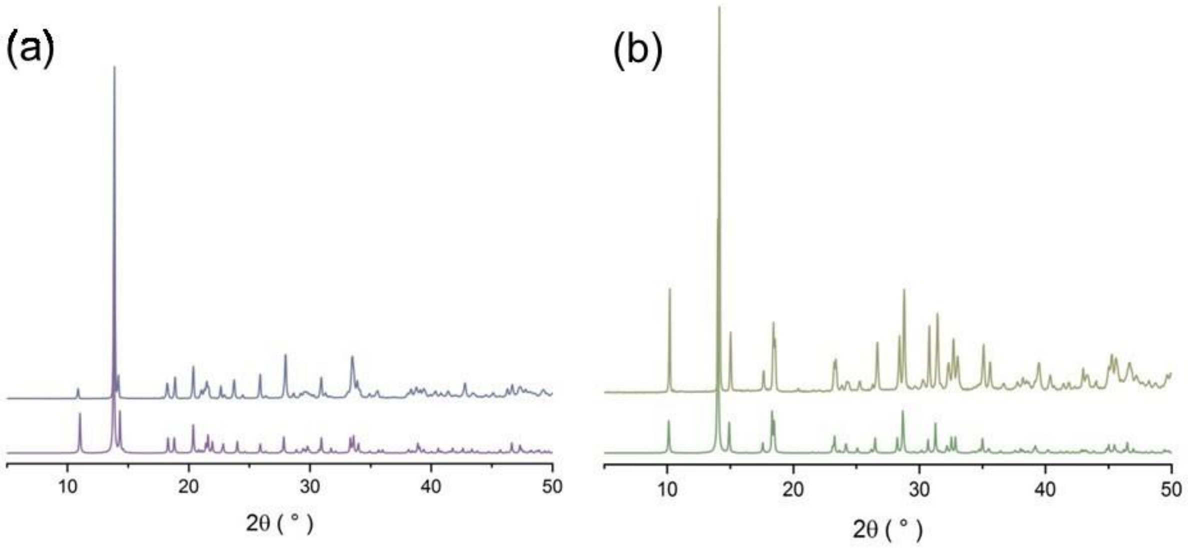

Figure S7. Powder X-ray diffractograms of (a) CALF-20 and (b) CALF-15 products. Measured patterns – top, calculated patterns – bottom.

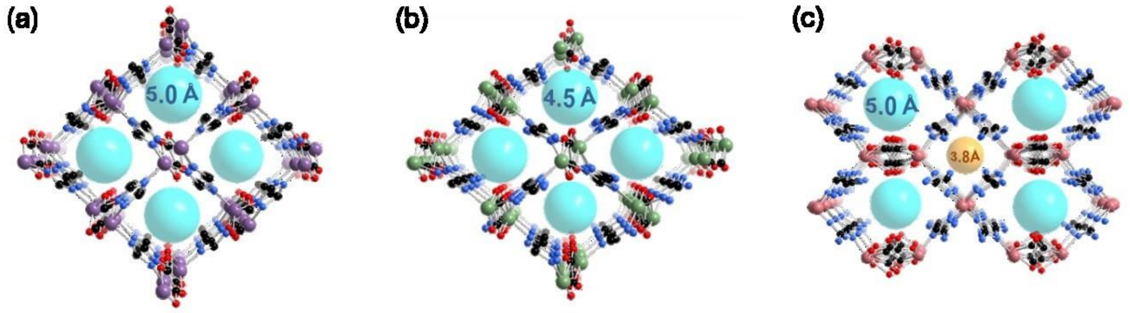

Figure S8. Scheme of the frameworks oriented along (100) for (a) CALF-20, (b) CALF-15 and (001) for (c) NICS-24 micropore channels. Spheres located within the channels symbolize free space with the indicated estimated pore determined from the distances between opposite atoms defining the channel boundaries.

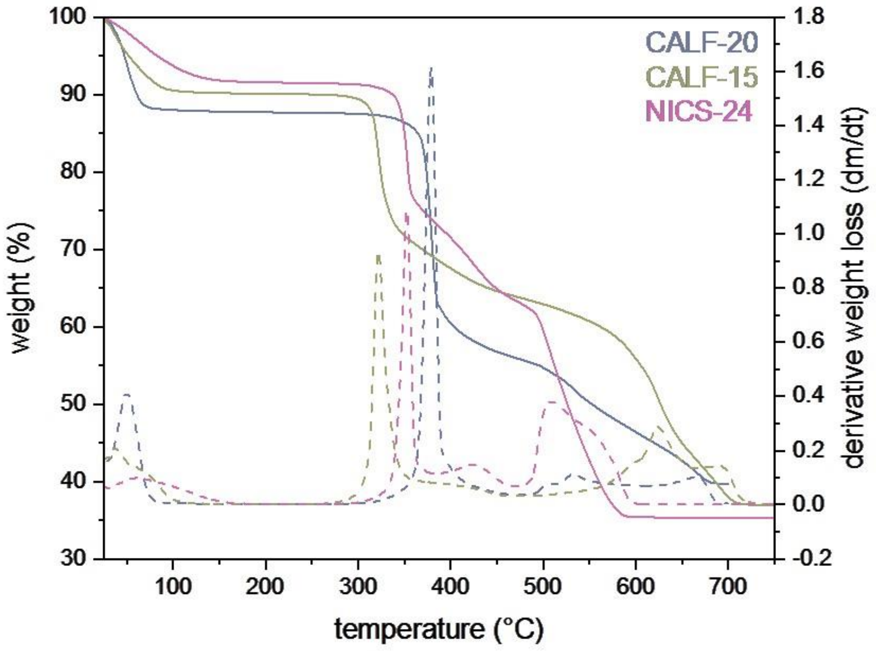

Figure S9. TG curves of investigated materials (full lines) and with the corresponding DTG curves (dashed lines).

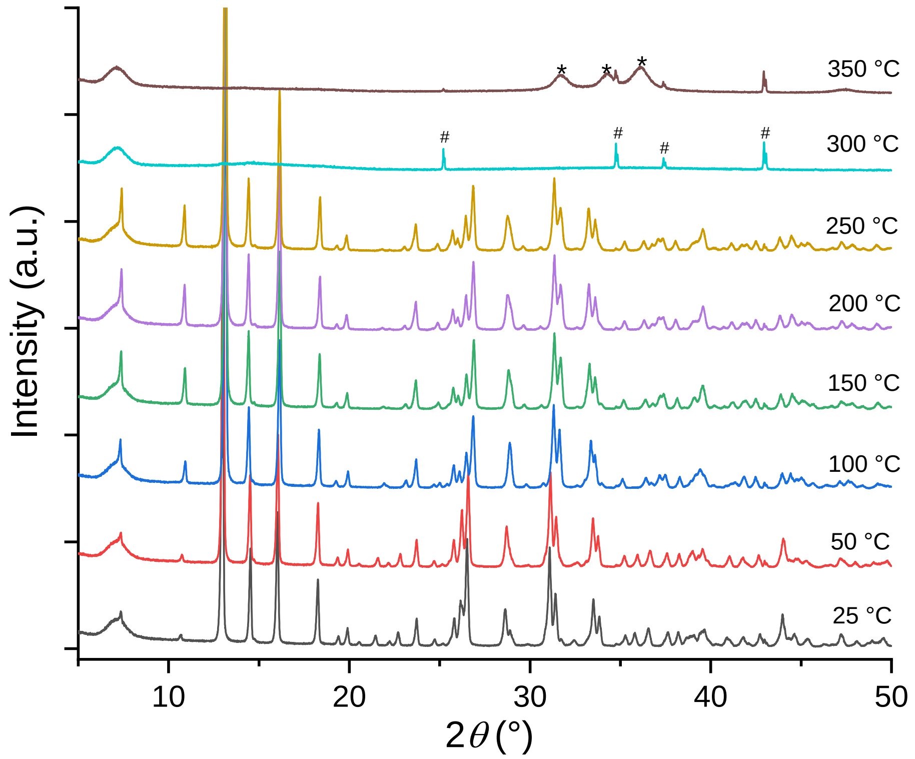

Figure S10. Temperature-programmed XRD patterns for NICS-24 measured at specified temperatures. Peaks marked with a number signs and asterisks are assigned to unknown phase and ZnO respectively.

Table S5. Comparison of unit cell parameters for NICS-24; RT - as-synthesized, 250 – thermally treated at 250 °C.

	NICS-24-RT	NICS-24-250
Space group	$P2_1/c$ (no. 14)	$P2_1/c$ (no. 14)
a (Å)	8.5703(3)	8.4138(3)
b (Å)	23.9768(9)	24.006(1)
c (Å)	7.3145(3)	7.3865(2)
β (°)	104.892(2)	104.208(2)
V (Å^3)	1452.6(1)	1446.33(9)

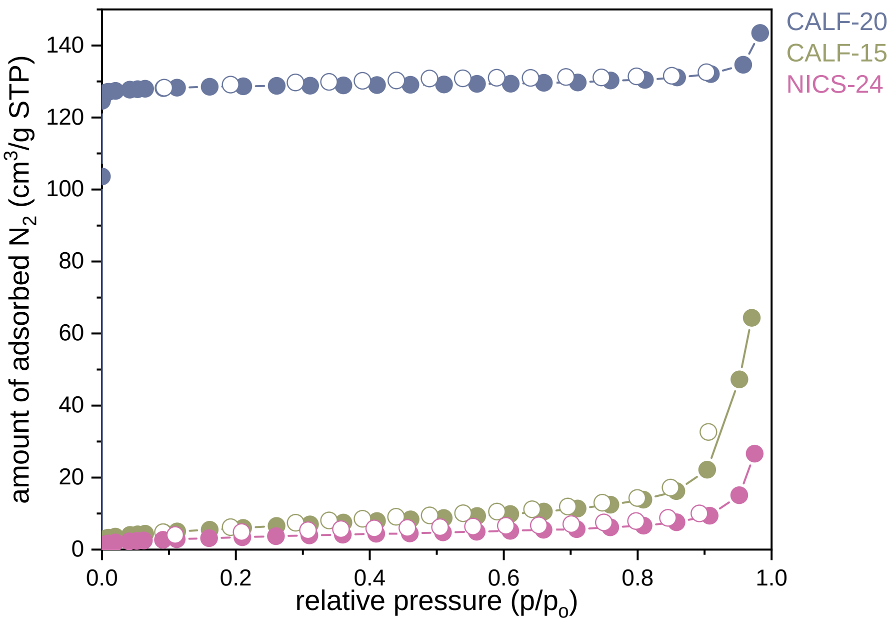

Figure S11. N_2 isotherms of investigated materials measured at 77 K. Adsorption points – full circles, desorption points – empty circles.

4. Sorption properties

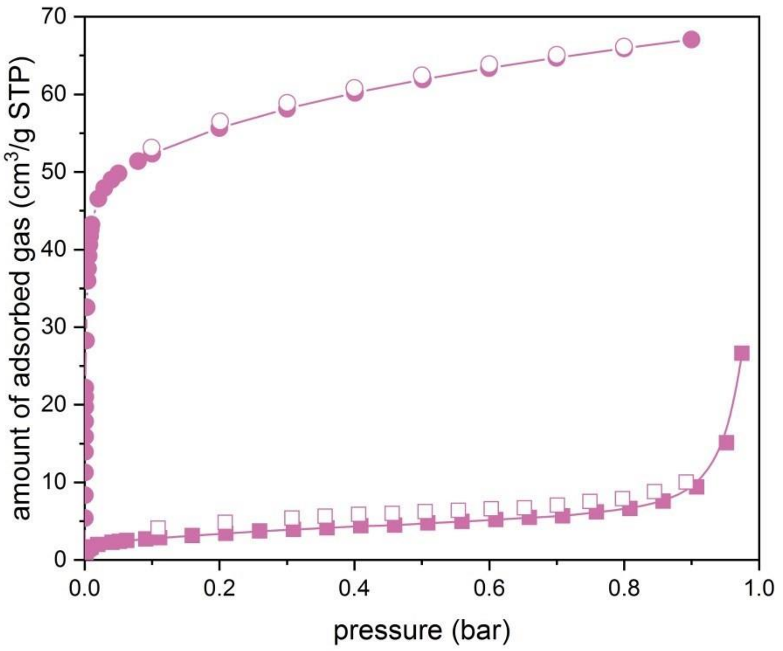

Figure S12. Comparison of sorption isotherms for N_2 measured at 77 K (squares) and for CO_2 measured at 273 K (circles) for NICS-24 material. Full symbols – adsorption, empty symbols – desorption.

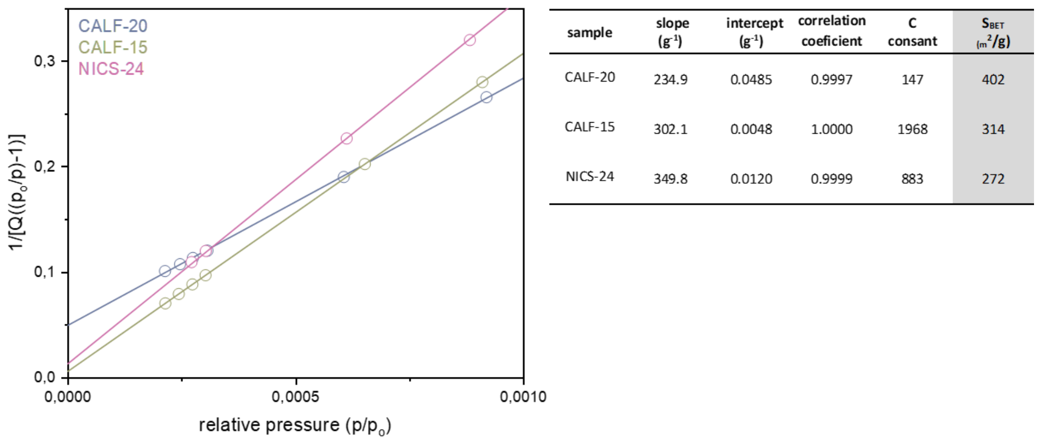

sample	slope (g^{-1})	intercept (g^{-1})	correlation coeficient	C consant	S_{BET} (m^2/g)
CALF-20	234.9	0.0485	0.9997	147	402
CALF-15	302.1	0.0048	1.0000	1968	314
NICS-24	349.8	0.0120	0.9999	883	272

Figure S13. BET plots based on CO_2 isotherms measured at 273 K for investigated materials. Parameters of linear fit (lines) and calculated BET values are enlisted in the table.

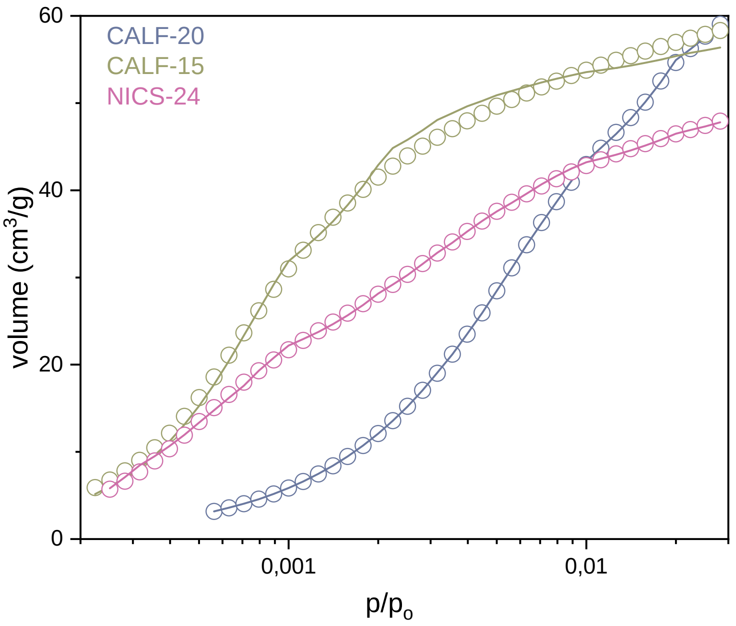

Figure S14. NLDFT fit plots based on CO_2 isotherms measured at 273 K for investigated materials. Measured data (circles) fitted data (full lines).

Table S6. (a) Representative amine-appended MOFs utilized for direct air capture. (b) MOFs with amine-decorated pores reported as (potential) adsorbents for pre- or post-combustion CO_2 capture and separation from gas mixtures.

(a)

MOF	CO_2 uptake at 298 K (mmol g^{-1}/400 ppm)	Q_{ST} (kJ mol^{-1})	ref.
mmen-Mg_2(dobpdc)	2.00 (390 ppm)	71	[15]
en-Mg_2(dobpdc)	2.83 (390 ppm)	49–51	[16, 17]
men-Mg_2(dobpdc)	0.41	65–77	[18]
nmen-Mg_2(dobpdc)	0.76	60–72	[19]
pn-Mg_2(dobpdc)	1.76	/	[20]
nmpn-Mg_2(dobpdc)	1.76	/	[20]
MIL-101(Cr)-TREN	0.35	/	[21]
MIL-101(Cr)-SO_3H-TAEA	1.12	87	[22]
en-Mg-MOF-74	1.51	/	[16]

(b)

MOF	CO_2 uptake[a] (mmol g^{-1})	Q_{ST} (kJ mol^{-1})	ref.
CALF-15[b]	2.05 (298 K, 0.15 bar)	40.8	[23, 24]
CALF-15[c]	2.70 (303 K, 0.15 bar)	46	[24]
Cu(adci)-2	2.01 (298 K, 0.15 bar)	27.5	[25]
ZnF(daTZ)	0.96 (298 K, 0.15 bar)	33	[26]
MUF-16	1.10 (298 K, 0.15 bar)	32.3	[27]
MUF-17	1.58 (298 K, 0.15 bar)	28.3	[28]
bio-MOF-11	6.06 (298 K, 1 bar)	31.7	[29]
MIL-53(Al)-NH_2	2.30 (288 K, 5 bar)	38.4	[30]
UiO-66-NH_2	1.15 (298 K, 0.15 bar)	28	[31]

[a]CO_2 uptake values were extracted from the lowest pressure data point reported in the literature, when such data was provided.

CCDC reference code: [b]734994 and [c]1428296

abbreviations	
dobpdc	4,4′-dioxido-3,3′-biphenyldicarboxylate
mmen	*N*,*N*′-dimethylethylenediamine
en	ethylenediamine
men	1-methylethyl-enediamine
nmpn	*N*-methyl-1,3-diaminopropane
pn	1,3-diaminopropane
nmen	*N*-methylethylenediamine
TREN or TAEA	tris(2-aminoethyl)amine
adci	2-amino-4,5-dicyanoimidazolate
daTZ	3,5-diamino-1,2,4-triazolate

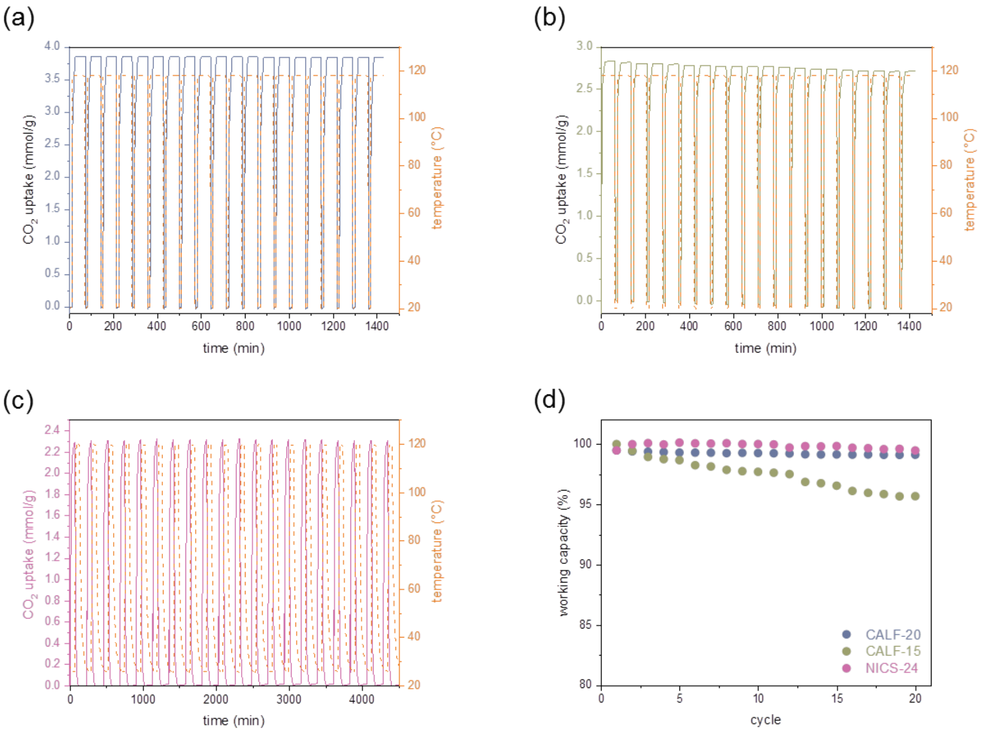

Figure S15. CO_2 adsorption cycling with temperature swing adsorption (TSA) regeneration for (a) CALF-20, (b) CALF-15 and (c) NICS-24. (d) Loss of working adsorption capacity during TSA regeneration cycles for corresponding materials.

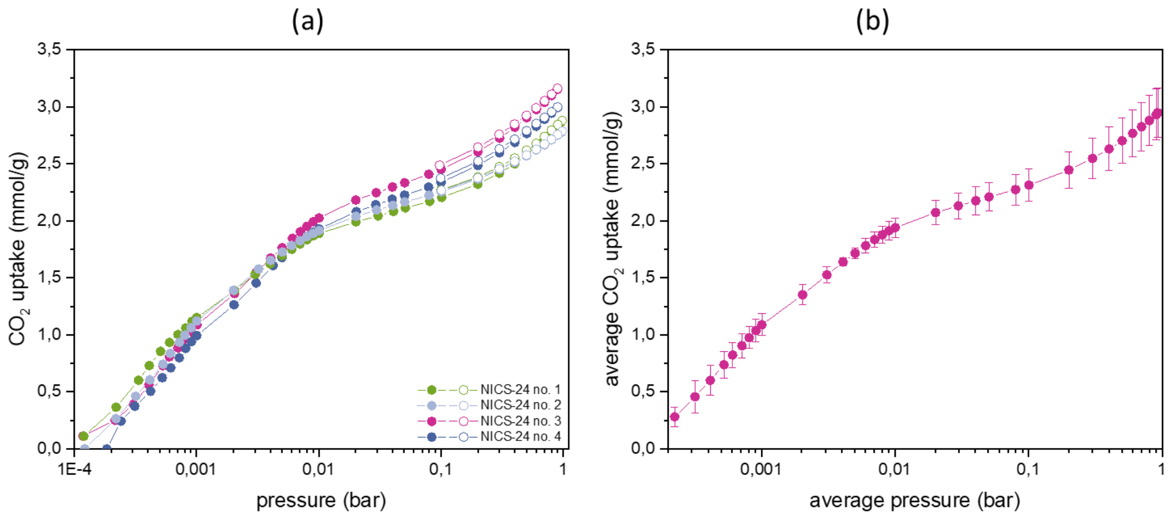

Figure S16. (a) CO_2 isotherms of four batches of NICS-24 measured at 273 K. Full circles – adsorption, empty circles – desorption. (b) Averaged adsorption isotherm with indicated error bars based on obtained data for four batches of product. The batch-to-batch variation in CO_2 uptake arises from minor differences can be due variations in diffusivity. The error bars in the region near CO_2 saturation (approximately 0.01 bar) are the smallest, indicating consistent micropore accessibility and structural integrity across batches. Variability becomes more pronounced at higher pressures, likely due to morphological differences (e.g., agglomeration or crystallite size) rather than deviations in the framework structure or sorption site properties.

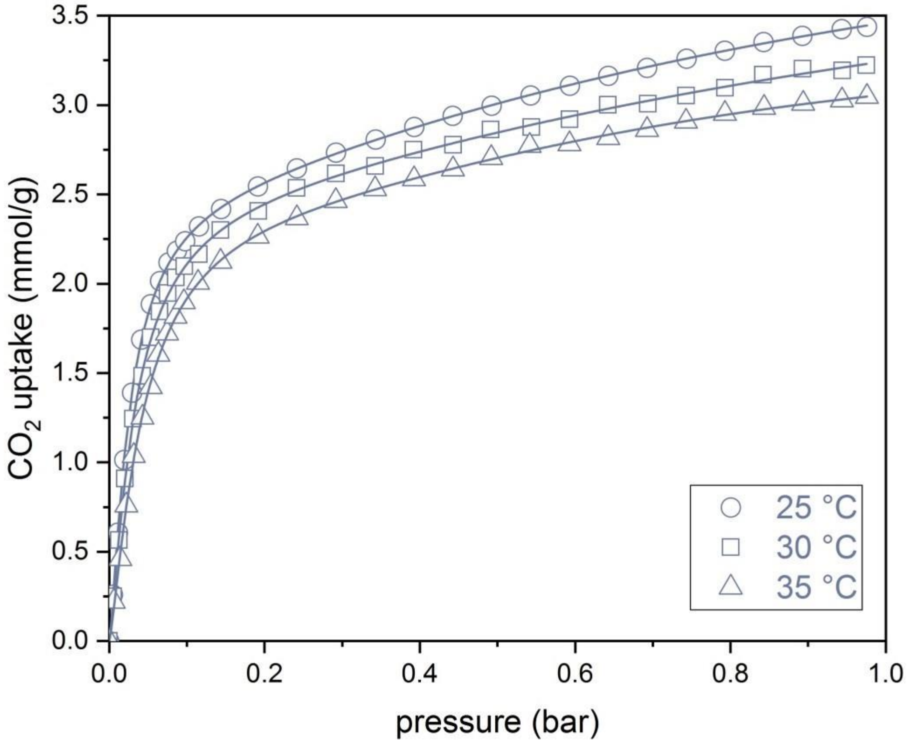

Figure S17. CO_2 isotherms measured at specified temperatures for CALF-20. Symbols – measured points, lines – Dual Site Langmuir model fit.

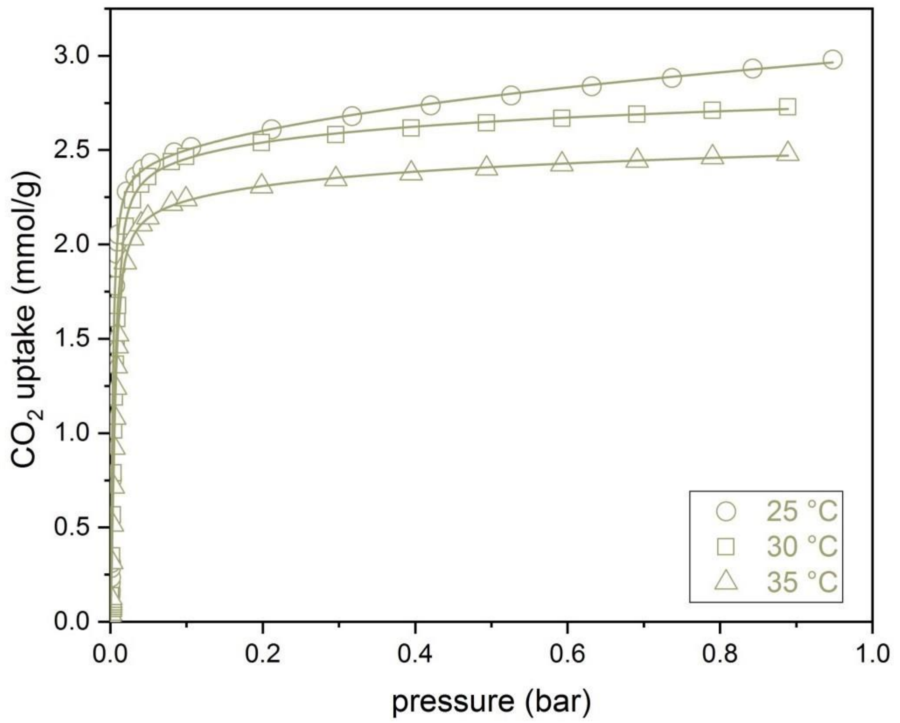

Figure S18. CO_2 isotherms measured at specified temperatures for CALF-15. Symbols – measured points, lines – Dual Site Langmuir model fit.

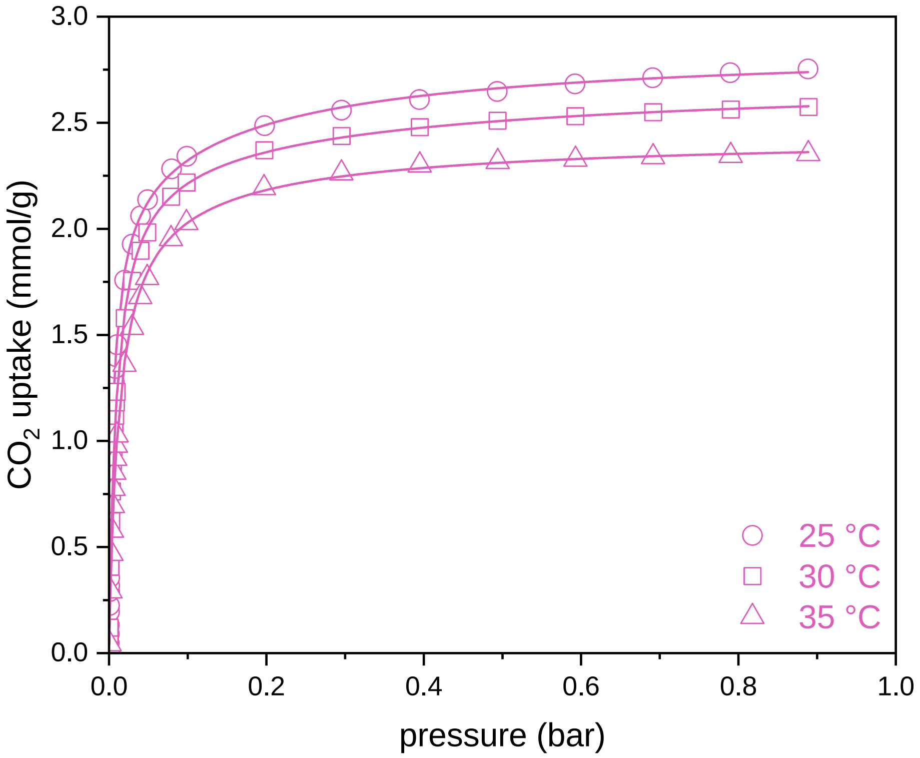

Figure S19. CO_2 isotherms measured at specified temperatures for NICS-24. Symbols – measured points, lines – Dual Site Langmuir model fit.

Table S7: Fitting parameters for Dual Site Langmuir isotherm model.

sample	q_{m1}	K_1	q_{m2}	K_2	R^2
**CALF-20 (25 °C)**	2.71 ± 0.01	38.0 ± 0.3	15 ± 4	0.06 ± 0.02	0.99889
**CALF-20 (30 °C)**	2.71 ± 0.01	29.5 ± 0.2	4.8 ± 0.7	0.15 ± 0.0.02	0.99849
**CALF-20 (35 °C)**	2.67 ± 0.01	21.9 ± 0.2	6 ± 2	0.09 ± 0.03	0.99857
**CALF-15 (25 °C)**	2.6 ± 0.1	443 ± 53	31 ± 2	0.03 ± 0.02	0.9778
**CALF-15 (30 °C)**	2.5 ± 0.3	231 ± 53	0.4 ± 0.1	0.5 ± 0.1	0.98957
**CALF-15 (35 °C)**	2.6 ± 0.1	118 ± 14	1.0 ± 0.1	0.2 ± 0.1	0.98554
**NICS-24 (25 °C)**	0.68 ± 0.06	6 ± 2	2.18 ± 0.08	193 ± 13	0.9987
**NICS-24 (30 °C)**	2.3 ± 0.2	107 ± 12	0.4 ± 0.2	2.8 ± 0.5	0.9952
**NICS-24 (35 °C)**	0.3 ± 0.2	15 ± 5	2.2 ± 0.3	81 ± 14	0.9944

q_{m1} and q_{m2} - maximum adsorption capacities for sites 1 and 2 respectively. K_1 *and* K_2 - Langmuir constants for sites 1 and 2 respectively, R^2 – proportion of variance.

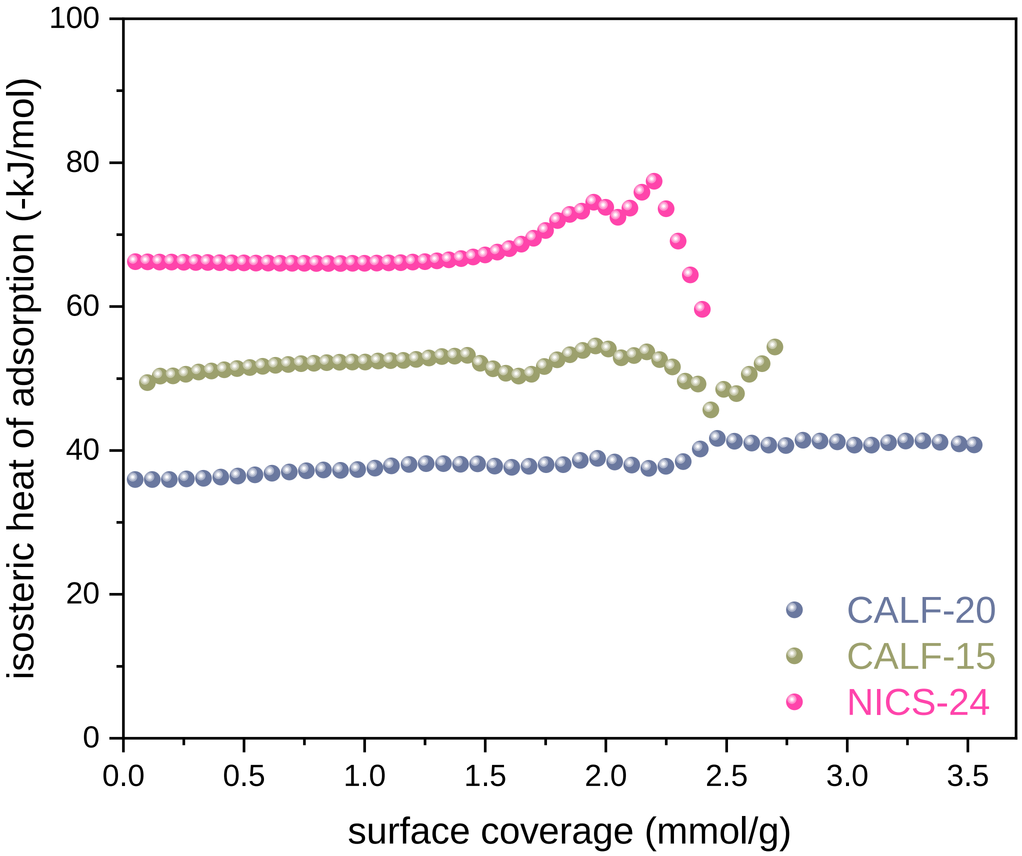

Figure S20. Isosteric heat of adsorption of investigated materials calculated using Clausius-Clapeyron equation for the entire surface coverage range valid for the individual sample. Fluctuations observed for Q_{st} curves at higher coverages approaching saturation region (for NICS-24 in particular) can introduce non-ideal interactions that Clausius-Clapeyron principal does not account for. This may arise from multilayer adsorbate-adsorbate interactions.

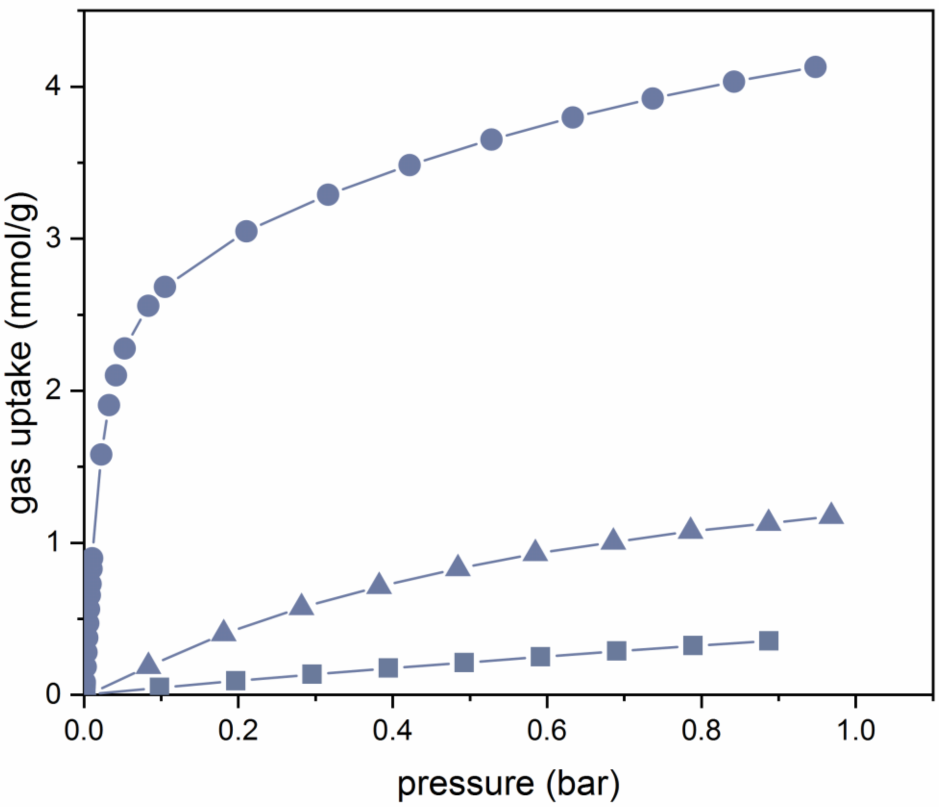

Figure S21. Comparison of adsorption isotherms for single gas components measured at 25 °C for CALF-20. Circles - CO_2, squares – N_2, triangles – O_2.

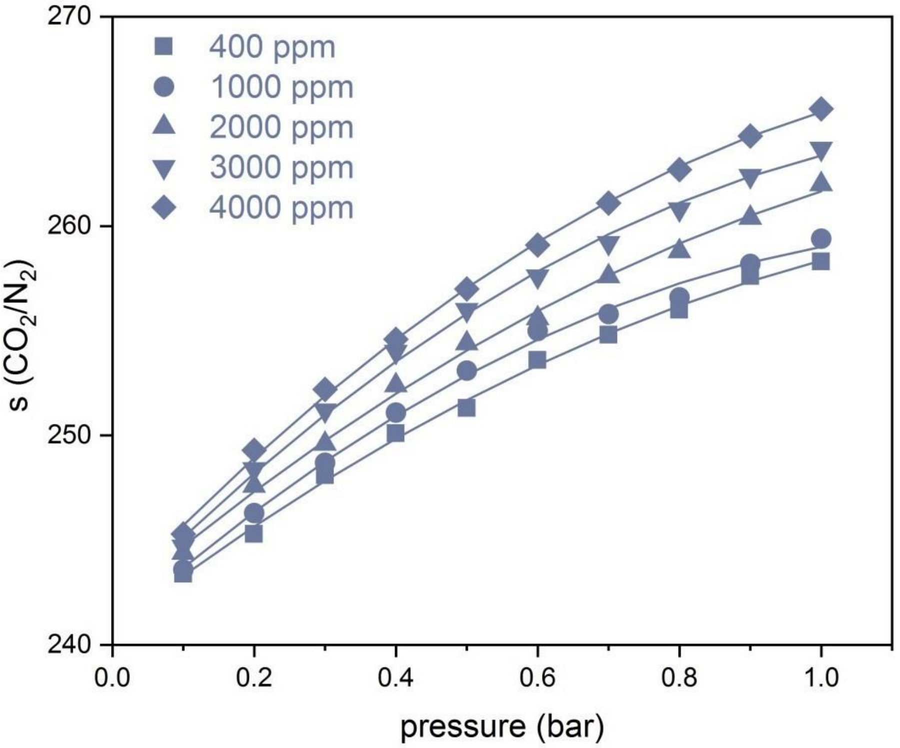

Figure S22. IAST CO_2/N_2 selectivity at specified CO_2/N_2 pressure ratios for CALF-20.

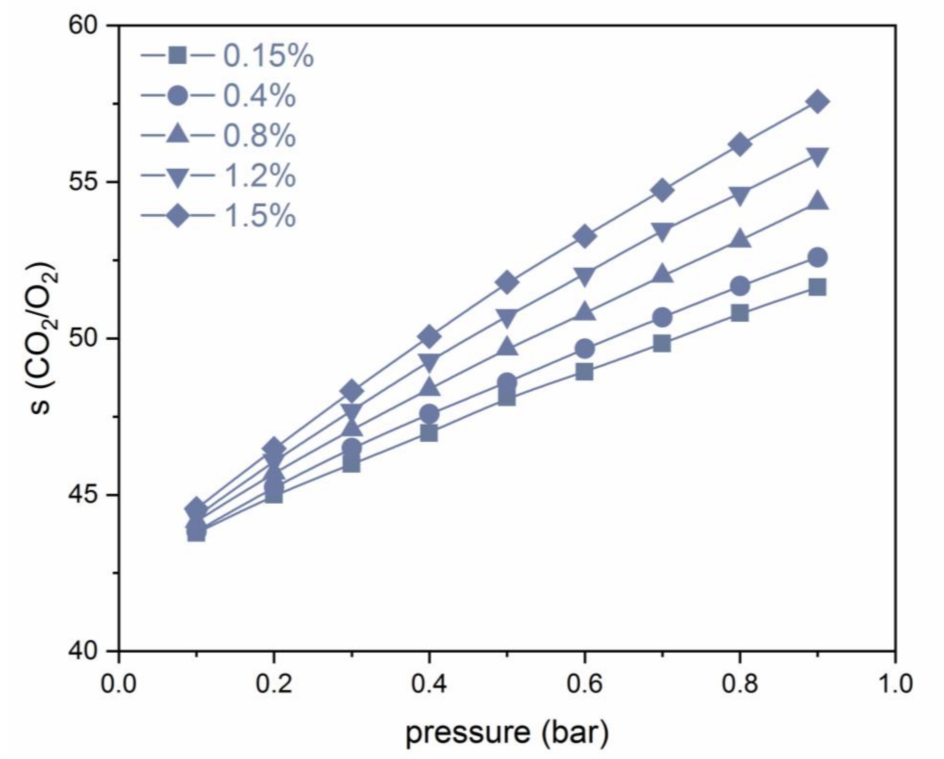

Figure S23. IAST CO_2/O_2 selectivity at specified CO_2/O_2 pressure ratios for CALF-20.

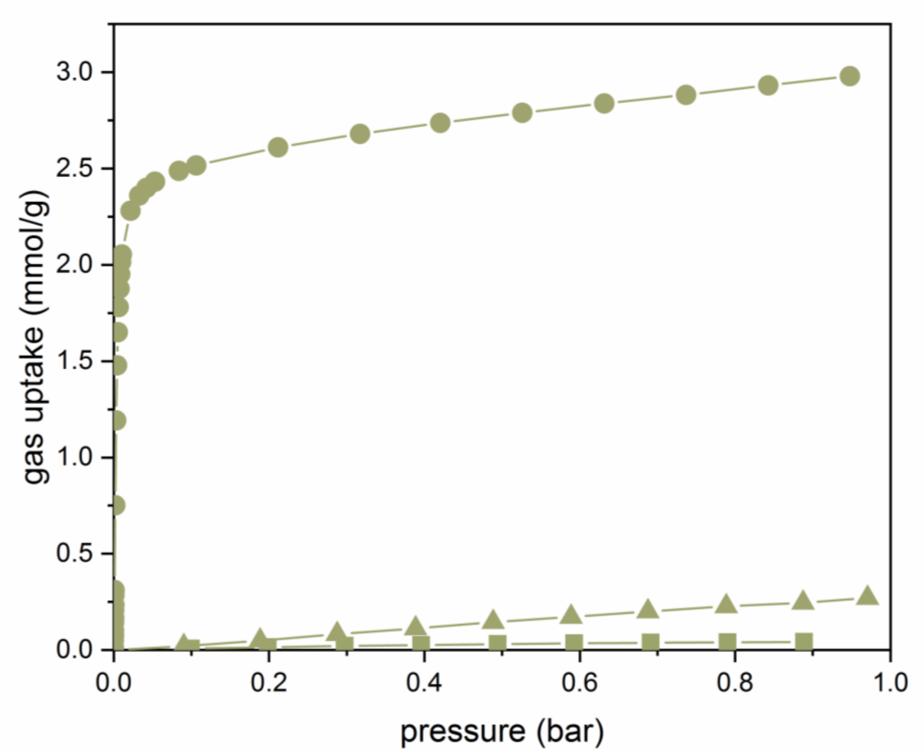

Figure S24. Comparison of adsorption isotherms for single gas components measured at 25 °C for CALF-15. Circles - CO_2, squares – N_2, triangles – O_2.

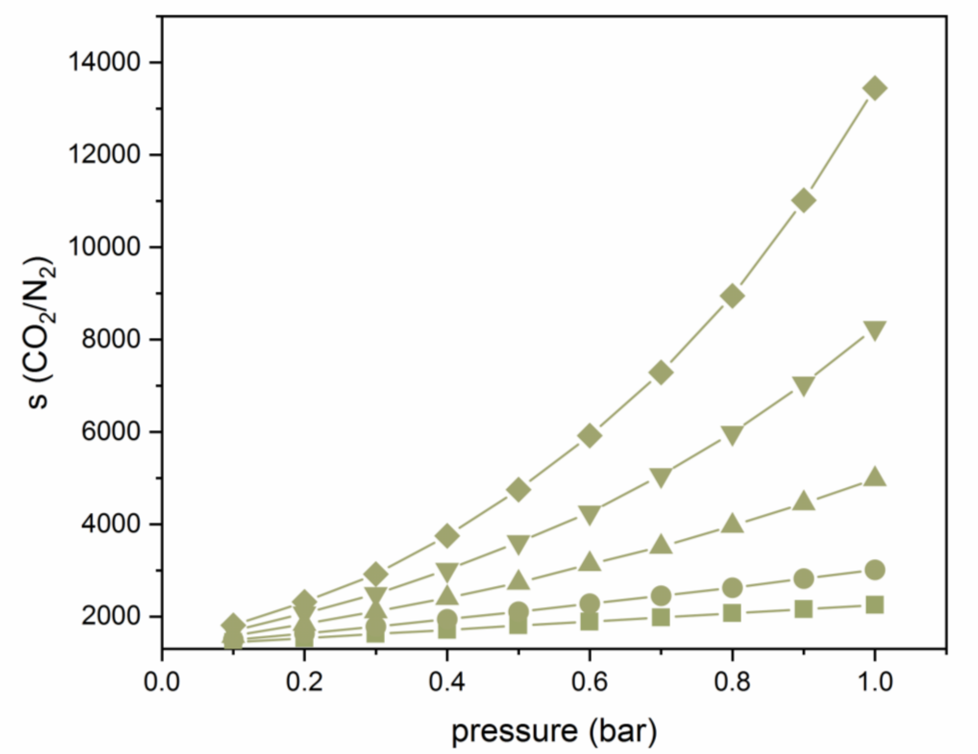

Figure S25. IAST CO_2/N_2 selectivity at specified CO_2/N_2 pressure ratios for CALF-15.

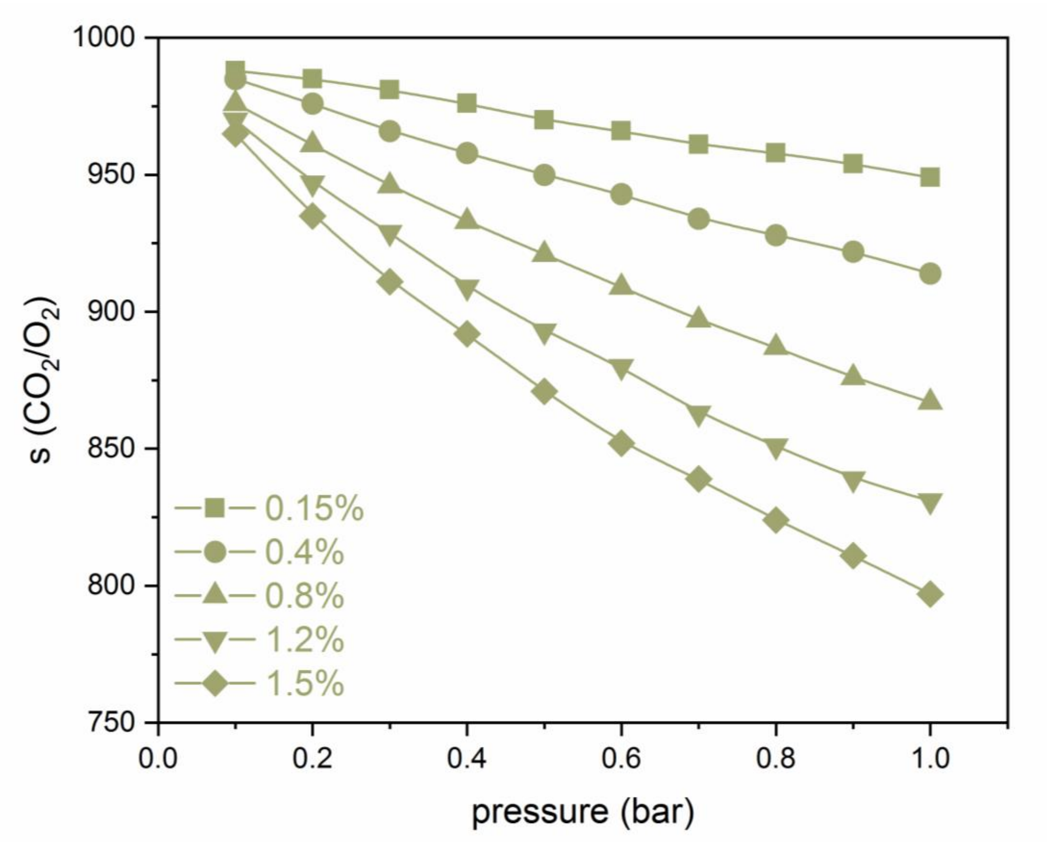

Figure S26. IAST CO_2/O_2 selectivity at specified CO_2/O_2 pressure ratios for CALF-15.

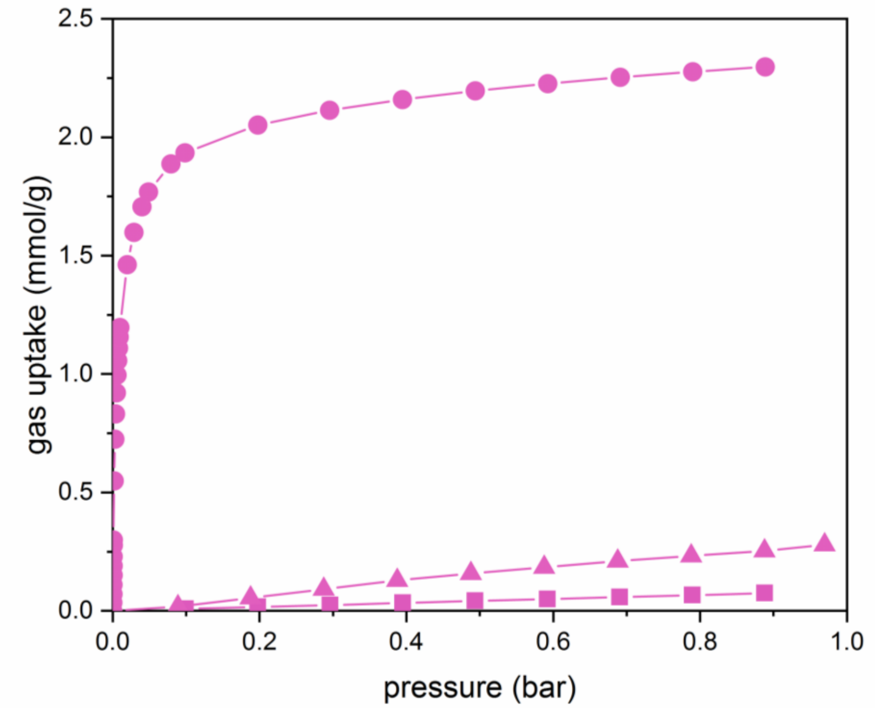

Figure S27. Comparison of adsorption isotherms for single gas components measured at 25 °C for NICS-24. Circles - CO_2, squares – N_2, triangles – O_2.

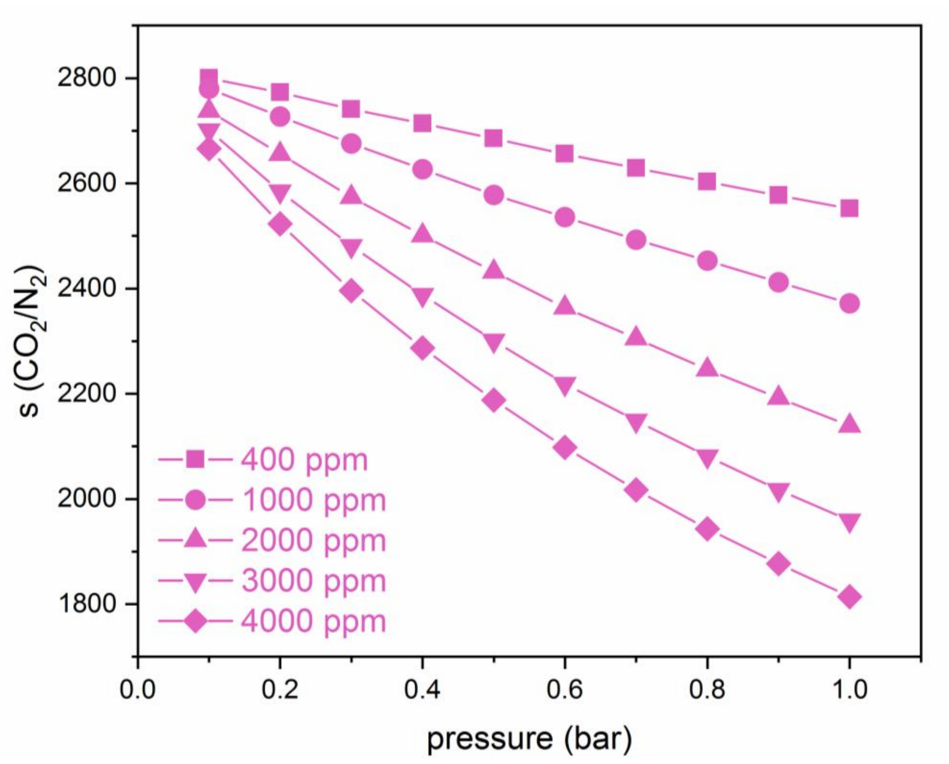

Figure S28. IAST CO_2/N_2 selectivity at specified CO_2/N_2 pressure ratios for NICS-24.

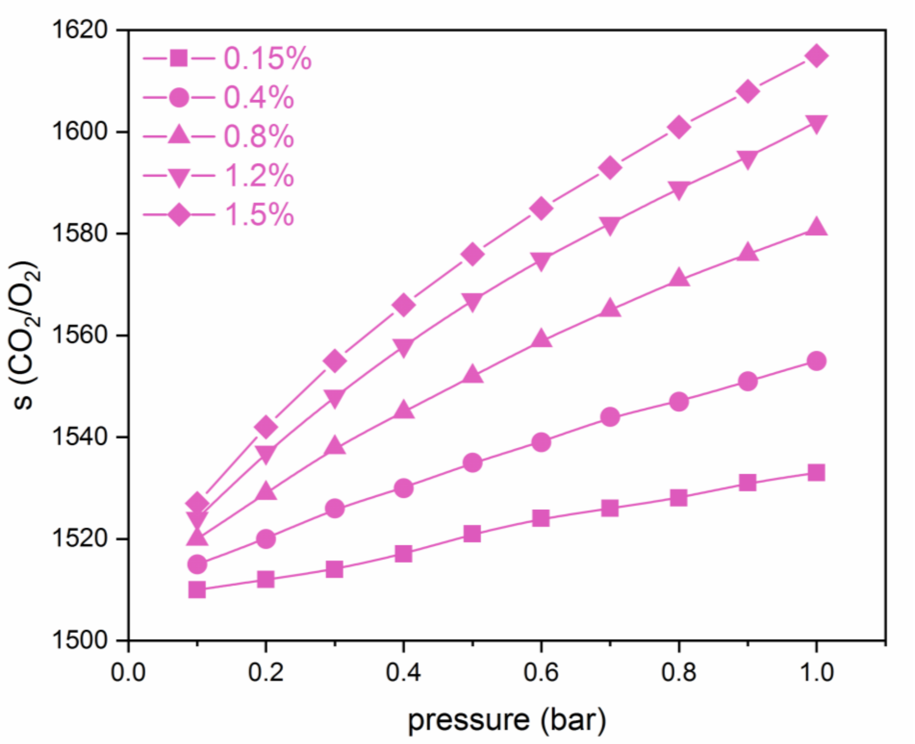

Figure S29. IAST CO_2/O_2 selectivity at specified CO_2/O_2 pressure ratios for NICS-24.

Table S8. Unit cell parameters of parent and H_2O soaked NICS-24 samples obtained by Rietveld refinement.

sample	*a* / Å	*b* / Å	*c* / Å	*β* / °	*V* / Å³
as-synth	8.55584	24.00745	7.31037	104.8885	1451.1667
RT-3d	8.55152	23.99483	7.30818	104.8830	1449.2760
RT-7d	8.55247	23.99531	7.30752	104.8879	1449.2999
60-1d	8.55379	23.99833	7.30934	104.8845	1449.5275
60-3d	8.55286	23.99631	7.30805	104.8886	1450.0891

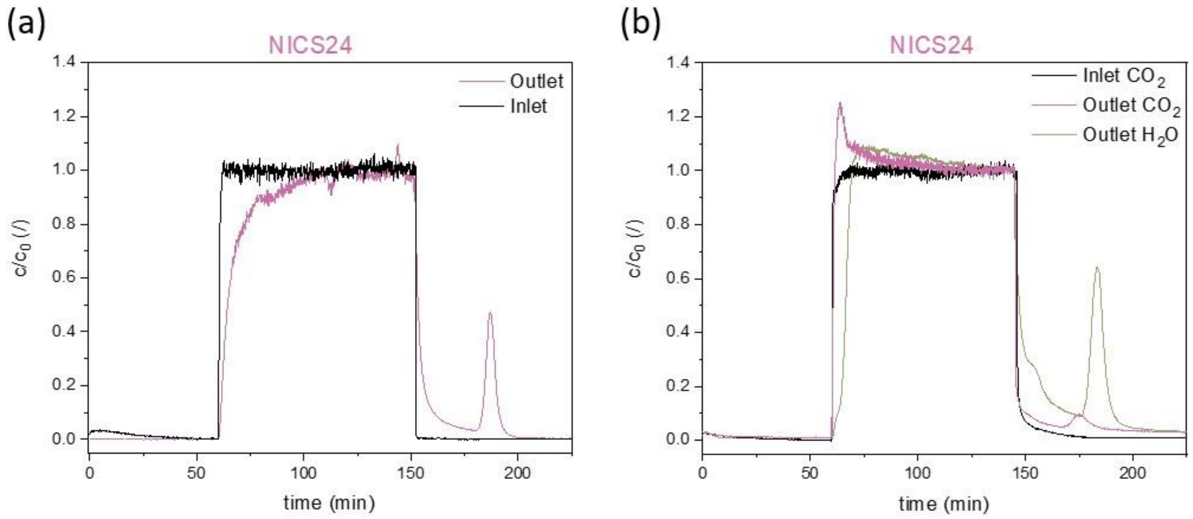

Figure S30. The dynamic breakthrough curve measurement for NICS-24 at 25°C under (a) dry conditions and (b) humid conditions (50%RH, 2000 ppm CO_2). The 'Inlet' and 'Outlet' refer to the inlet and outlet CO_2 concentration of the fixed-bed column.

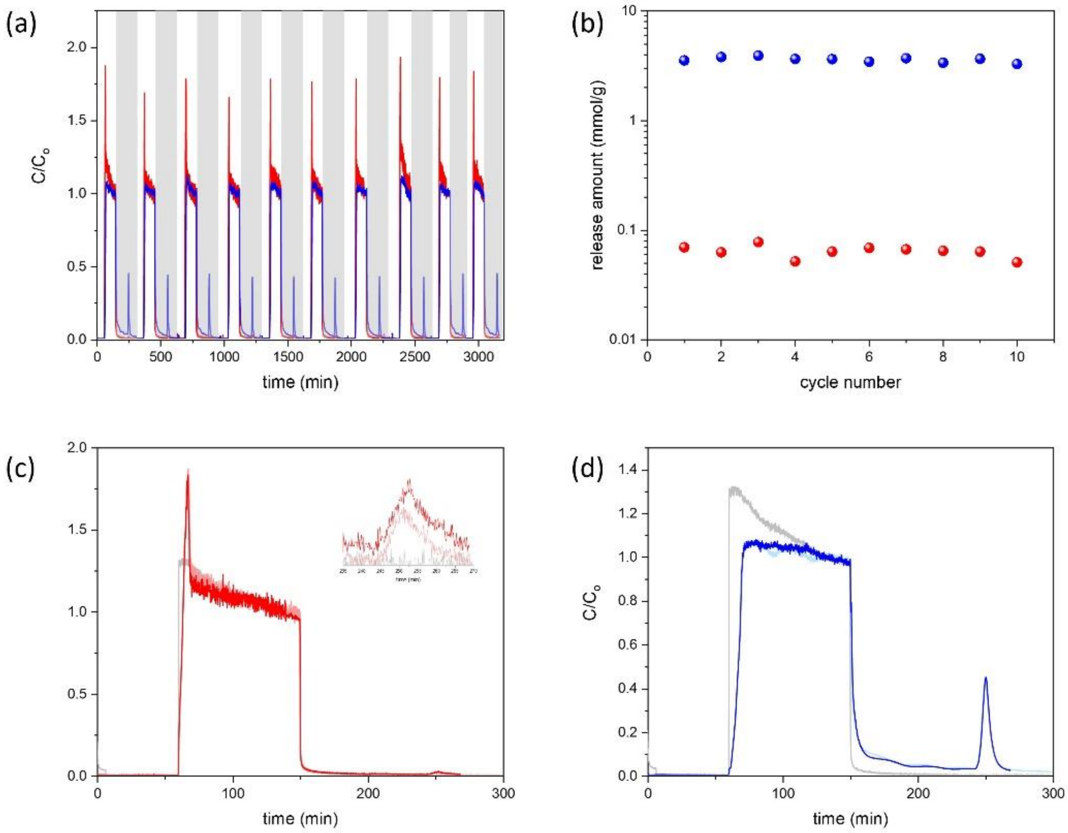

Figure S31. (a) Dynamic breakthrough curve measurement for NICS-24 at 25°C under humid conditions (50%RH, 2000 ppm CO_2) for 10 sequential cycles. Blue line – CO_2 response, red line – H_2O response. Grey areas represent desorption steps – He purge at 120 °C. (b) Amount of the released CO_2 (red dots) and H_2O (blue dots) during 10 desorption steps. (c) Comparison of breakthrough curves for CO_2 1st cycle (light red) and 10th cycle (dark red). Inset shows zoomed time range where CO2 release response occurs. Grey line - blank cell response. (d) Comparison of breakthrough curves for H_2O for 1st cycle (light blue) and 10th cycle (dark blue). Grey line - blank cell response.

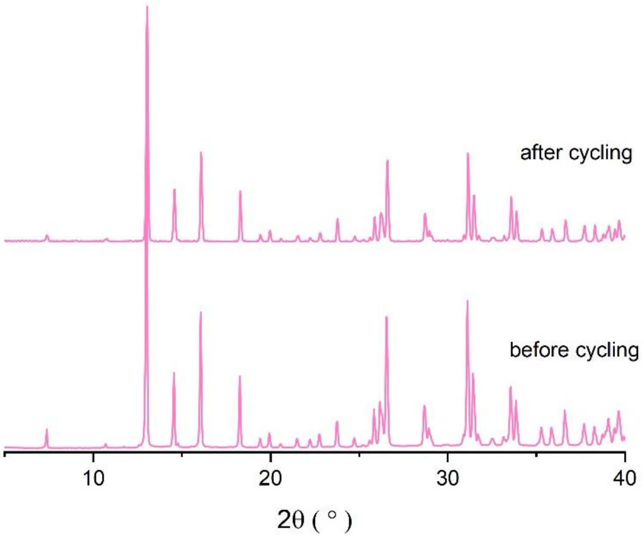

Figure S32. XRD patterns of NICS-24 before and after 10 cycles of dynamic breakthrough experiments under humid conditions.

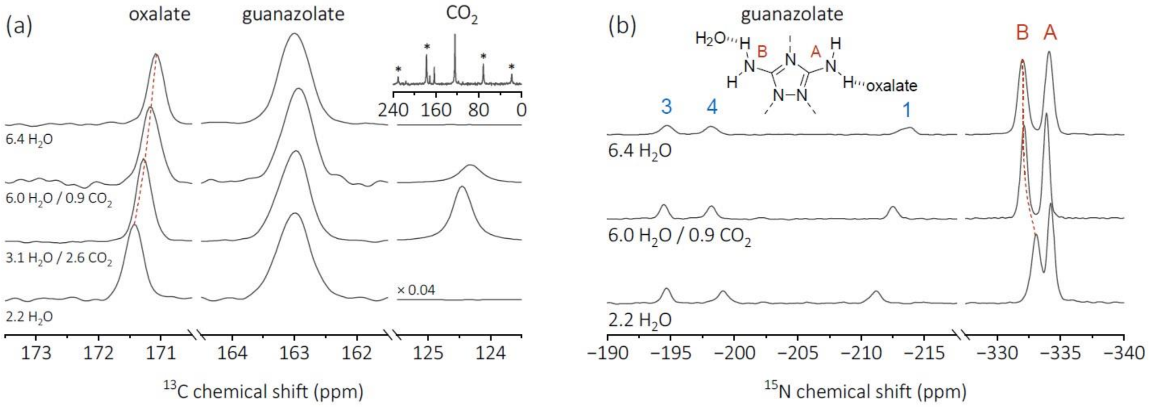

Figure S33. (a) ^{13}C MAS NMR spectra of NICS-24 with varying H_2O and CO_2 compositions. The inset highlights the extent of CO_2 sidebands in the ^{13}C MAS spectrum of NICS-24 containing 6 H_2O and 0.9 CO_2 molecules per unit cell, recorded at a sample spinning frequency of 8 kHz. (b) ^{1}H-^{15}N CP-MAS spectra of selected NICS-24 samples. The red dashed lines in both stack plots act as guides to illustrate the chemical shift dependence on channel B content. Hydrogen bonds slightly shift these peaks, and one might expect separate peaks for bonded and non-bonded NH_2 groups and oxalates. However, due to the fast dynamics of the guest molecules, only a single peak at the averaged chemical shift position is observed. The observed shifting reflects the changing ratio between the bonded and non-bonded states.

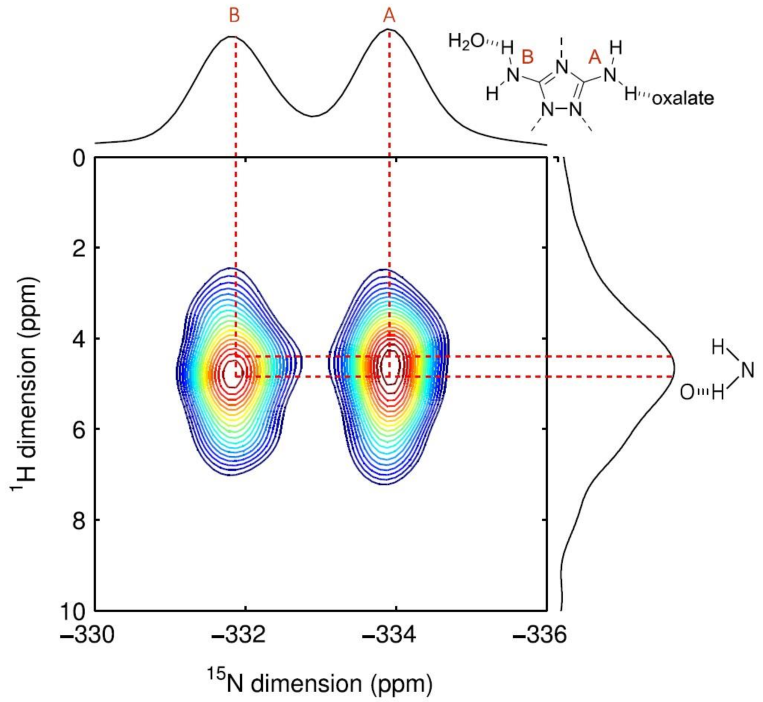

Figure S34. ^{1}H-^{15}N CP-HETCOR NMR spectrum of NICS-24 containing 6.4 H_2O molecules per unit cell. The ^{15}N signals from the NH_2 groups correlate with protons not involved in hydrogen bonding, as well as with protons involved in hydrogen bonding, which are slightly shifted downfield.

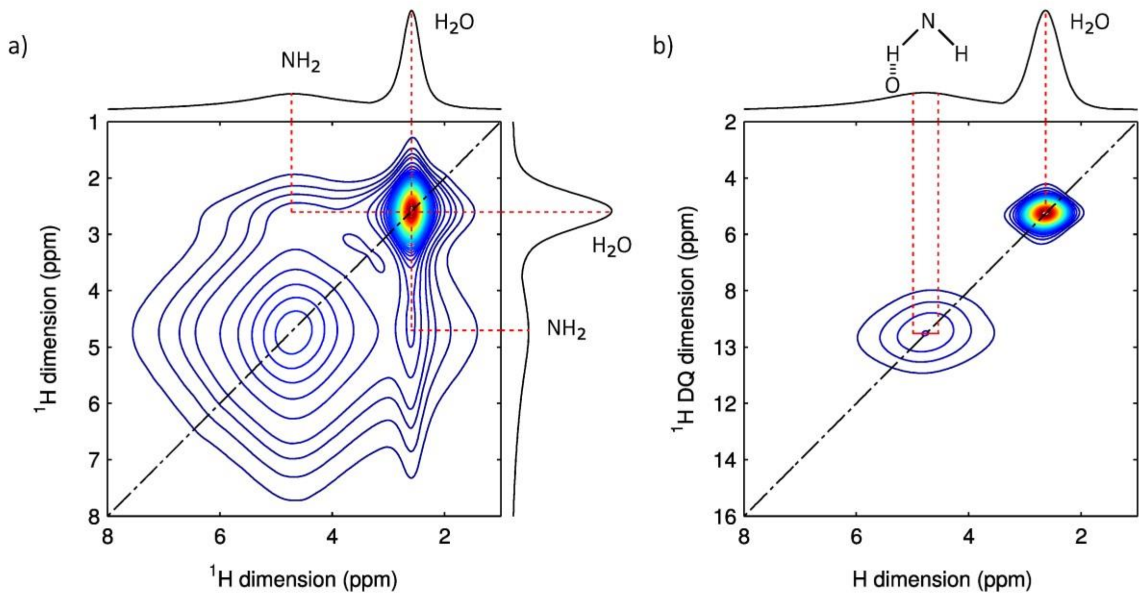

Figure S35. (a) ^1H-^1H single-quantum to single-quantum (SQ-SQ) and (b) ^1H-^1H double-quantum to single-quantum (DQ-SQ) NMR spectra of NICS-24 with 6.4 H_2O molecules per unit cell. In (a), the dashed lines indicate correlations between H_2O and NH_2 groups, while in (b), the dashed lines highlight correlations between equivalent protons of H_2O and slightly shifted protons within the same NH_2 groups.

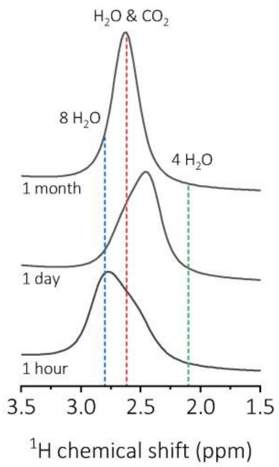

Figure S36. ^1H MAS NMR spectra of NICS-24 containing 6 H_2O and 0.9 CO_2 molecules per unit cell, recorded at three different time points: immediately after preparation (bottom), one day later (middle), and one month after being sealed in the NMR rotor (top). The green, red, and blue dashed lines indicate the chemical shift positions corresponding to half-hydrated, fully-hydrated, and CO_2-water mixed phases, respectively. Initially, phase segregation occurs with fully-hydrated domains appearing at the edges of the crystals and CO_2-H_2O domains spreading inwards. Over time, water redistributes slightly, resulting in an averaged peak representing both half-hydrated and fully-hydrated units. After several days, equilibrium is reached, resulting in a single peak basically at the position of CO_2-water mixed domains, demonstrating that this redistribution is energetically favourable.

4. References

[1] J.-B. Lin, T.T.T. Nguyen, R. Vaidhyanathan, J. Burner, J.M. Taylor, H. Durekova, F. Akhtar, R.K. Mah, O. Ghaffari-Nik, S. Marx, N. Fylstra, S.S. Iremonger, S. S., K.W. Dawson, P. Sarkar, P. Hovington, A. Rajendran, T.K. Woo, G.K.H: Shimizu, *Science* **2021**, *374*, 1464–1469.

[2] J. Peng, H. Wang, D.H. Olson, Z. Li, J. Li, *Chem. Commun.* **2017**, *53*, 9332–9335.

[3] G. Metz, X. L. Wu, S. O. Smith, *J. Mag. Res. A* **1994**, *110*, 219–227.

[4] A. E. Bennett, C. M. Rienstra, M. Auger, K. V. Lakshmi, R. G. Griffin, *J. Chem. Phys.* **1995**, *103*, 6951–6958.

[5] I. Scholz, P. Hodgkinson, B. H. Meier, M. Ernst*, J. Chem. Phys.* **2009**, *130*, 114510.

[6] M. Kotecha, N. P. Wickramasinghe, Y. Ishii, *Magn. Reson. Chem.* **2007**, *45*, S221–S230.

[7] M. Hohwy, H. J. Jakobsen, M. Edén, M. H. Levitt, N. C. Nielsen, *J. Chem. Phys.* **1998**, *108*, 2686–2694.

[8] M. Bak, J. T. Rasmussen, N. C. Nielsen, *J. Magn. Reson. A* **2000**, *147*, 296–330.

[9] A. E. Bennett, C. M. Rienstra, J. M. Griffiths, W. Zhen, P. T. Lansbury Jr., R. G. Griffin, *J. Chem. Phys.* **1998**, *108*, 9463–9479.

[10] M. Shen, B. Hu, O. Lafon, J. Trébosc, Q. Chen, J.-P. Amoureux, *J. Magn. Reson. A* **2012**, *223*, 107–119.

[11] R. Zhang, Y. Nishiyama, P. Sun, A. Ramamoorthy, *J. Magn. Reson. A* **2015**, *252*, 55–66.

[12] M. Feike, D. E. Demco, R. Graf, J. Gottwald, S. Hafner, H. W. Spiess, *J. Magn. Reson. A* **1996**, *122*, 214–221.

[13] A. Altomare, C. Cuocci, C. Giacovazzo, A. Moliterni, R. Rizzi, N. Correro, A. Falcicchio, *J. Appl. Crystallogr.* **2013**, *46*, 1231–1235.

[14] *TOPAS*, V.6.0; Bruker AXS, 2018.

[15] T. M. McDonald, W. R. Lee, J. A. Mason, B. M. Wiers, C. S. Hong, J. R. Long, *J. Am. Chem. Soc.* **2012**, 134, 7056–7065.

[16] S. Choi, T. Watanabe, T. H. Bae, D. S. Sholl, C. W. Jones, *J. Phys. Chem. Lett.* **2012**, *3*, 1136–1141.

[17] W. R. Lee, S. Y. Hwang, D. W. Ryu, K. S. Lim, S. S. Han, D. Moon, J. Choi, C. S. Hong, *Energy Environ. Sci.* **2014**, *7*, 744–751.

[18] H. Jo, W. R. Lee, N. W. Kim, H. Jung, K. S. Lim, J. E. Kim, D. W. Kang, H. Lee, V. Hiremath, J. G. Seo, H. Jin, D. Moon, S. S. Han, C. S. Hong, *ChemSusChem* **2017**, *10*, 541–550.

[19] W. R. Lee, J. E. Kim, S. J. Lee, M. Kang, D. W. Kang, H. Y. Lee, V. Hiremath, J. G. Seo, H. Jin, D. Moon, M. Cho, Y. Jung, C. S. Hong, *ChemSusChem* **2018**, *11*, 1694–1707.

[20] J. Park, J. R. Park, J. H. Choe, S. Kim, M. Kang, D. W. Kang, J. Y. Kim, Y. W. Jeong, C. S. Hong, *ACS Appl. Mater. Interfaces* **2020**, *12*, 50534–50540.

[21] L. A. Darunte, A. D. Oetomo, K. S. Walton, D. S. Sholl, C. W. Jones, *ACS Sustain. Chem. Eng.* **2016**, *4*, 5761–5768.

[22] H. Li, K. Wang, D. Feng, Y. P. Chen, W. Verdegaal, H. C. Zhou, *ChemSusChem* **2016**, *9*, 2832–2840.

[23] R. Vaidhyanathan, S. S. Iremonger, K. W. Dawson, G. K. H. Shimizu, *Chem. Commun.* **2009**, 5230–5232.

[24] A. Banerjee, S. Nandi, P. Nasa, R. Vaidhyanathan, *Chem. Commun.* **2016**, *52*, 1851–1854.

[25] D. Jo, S. K. Lee, K. H. Cho, J. W. Yoon, U. H. Lee, *ACS Appl. Mater. Interfaces* **2022**, *14*, 56707–56714.

[26] Z. Shi, Y. Tao, J. Wu, C. Zhang, H. He, L. Long, Y. Lee, T. Li, Y. B. Zhang, *J. Am. Chem. Soc.* **2020**, *142*, 2750–2754.

[27] O. T. Qazvini, S. G. Telfer, *ACS Appl. Mater. Interfaces* **2021**, *13*, 12141–12148.

[28] O. T. Qazvini, S. G. Telfer, *J. Mater. Chem. A* **2020**, *8*, 12028–12034.

[29] J. An, S. J. Geib, N. L. Rosi, *J. Am. Chem. Soc.* **2010**, *132*, 38–39.

[30] S. Couck, J. F. M. Denayer, G. V. Baron, T. Rémy, J. Gascon, F. Kapteijn, *J. Am. Chem. Soc.* **2009**, *131*, 6326–6327.

[31] G. E. Cmarik, M. Kim, S. M. Cohen, K. S. Walton, *Langmuir* **2012**, *28*, 15606–15613.
